# Multiple Emitting Amphiphilic Conjugated Polythiophenes‐Coated CdTe QDs for Picogram Detection of Trinitrophenol Explosive and Application Using Chitosan Film and Paper‐Based Sensor Coupled with Smartphone

**DOI:** 10.1002/advs.201801467

**Published:** 2018-11-12

**Authors:** Salah M. Tawfik, Mirkomil Sharipov, Sarvar Kakhkhorov, Mohamed R. Elmasry, Yong‐Ill Lee

**Affiliations:** ^1^ Anastro Laboratory Department of Chemistry Changwon National University Changwon 51140 Republic of Korea; ^2^ Egyptian Petroleum Research Institute (EPRI) Nasr City Cairo 11727 Egypt

**Keywords:** amphiphilic polythiophene‐coated quantum dots, environmental water, paper‐based sensors, smartphones, 2,4,6‐trinitrophenol (TNP) explosive

## Abstract

Novel multiple emitting amphiphilic conjugated polythiophene‐coated CdTe quantum dots for picogram level determination of the 2,4,6‐trinitrophenol (TNP) explosive are developed. Four biocompatible sensors, cationic polythiophene nanohybrids (CPTQDs), nonionic polythiophene nanohybrids (NPTQDs), anionic polythiophene nanohybrids (APTQDs), and thiophene copolymer nanohybrids (TCPQDs), are designed using an in situ polymerization method, which shows highly enhanced fluorescence intensity and quantum yield (up to 78%). All sensors are investigated for nitroexplosive detection to provide a remarkable fluorescence quenching for TNP and the quenching efficiency reached 96% in the case of TCPQDs. The fluorescence of the sensors are quenched by TNP through inner filter effect, electrostatic, π−π, and hydrogen bonding interactions. Under optimal conditions, the detection limits of CPTQDs, NPTQDs, APTQDs, and TCPQDs are 2.56, 7.23, 4.12, and 0.56 × 10^−9^ m, respectively, within 60 s. More importantly, portable, cost effective, and simple to use paper strips and chitosan film are successfully applied to visually detect as little as 2.29 pg of TNP. The possibility of utilizing a smartphone with a color‐scanning APP in the determination of TNP is also established. Moreover, the practical application of the developed sensors for TNP detection in tap and river water samples is described with satisfactory recoveries of 98.02−107.50%.

## 1 Introduction

The determination of trace amounts of chemical explosives is a key challenge toward efforts to secure public places and monitor drinking and waste water. It was reported that the explosive power of 2,4,6‐trinitrophenol (TNP) is similar to a highly explosive compound TNT.^1^ Apart from its explosive nature, TNP is considered as a main toxic pollutant, which harshly affects soil and ground water and poses a significant health hazard because of its high solubility in water.^2^ Short‐term exposure to TNP causes eye and skin irritation, whereas long‐term exposure may cause damage to the kidneys and respiratory organs.^3^ Therefore, the development of rapid, sensitive, selective, and on‐site sensory systems for the detection of traces of TNP is of considerable current attention for both national security and environmental protection.

Several analytical methods have been used for the detection of nitroaromatics. However, most of them are expensive, less sensitive, time consuming, and complex to handle for on‐site detection.^3^, ^4^ In contrast, fluorescent sensors for the detection of nitroexplosive have attracted huge attention due to their simplicity, ultrasensitivity, fast response time, and extensive applicability.^3^, ^5^ Hence, various fluorescent sensors for explosives detection have been developed based on combined surface‐imprinting and paper‐based microfluidic chip techniques,^6^ manganese‐doped carbon quantum dots (QDs),^7^ nitrogen and sulfur co‐doped graphene QDs,^8^ boron nitride QDs,^9^ metal organic frameworks,^10^ hybrid CdTe QDs,^11^ and conjugated polymers (CPs).^12^ However, these methods still possess some disadvantages such as the use of organic solvents, low sensitivity and selectivity, and nonapplicability for on‐site detection. In addition, the biocompatibility of the previous reported materials has not been extensively examined. Therefore, preparation of various effective sensors for detection of TNP in different systems (aqueous medium and solid state) with excellent analytical performance is highly attractive and challenging.

CPs are at the forefront among several materials utilized in the field of sensing nowadays.^13^ CPs possess outstanding light‐harvesting properties and amplified fluorescence signal response causing by “molecular wire effect” that, as a result, makes them highly sensitive toward analytes.^14^ Among them amphiphilic polythiophenes have been widely employed as optical sensors.^15^ So far, few works have been reported for nitroexplosive detection based on CP probes.^12^, ^16^, ^17^ Nevertheless, low sensitivity and selectivity of these probes remain challenging due to the absence of suitable receptor, interferences by other electron deficient nitroaromatics, and less practicability in aqueous media and solid state detection. More importantly, the design of portable device for simple analysis of nitroexplosives has not been developed yet, which reduces the applicability of previously mentioned techniques.

QDs are tunable in size, photostable, and extremely effective fluorophores with a strong bandgap luminescence.^18^ Surface‐coated QDs are widely applied as fluorescent probes for various materials.^19^ However, the development of biocompatible and multiple emitting compounds by a facile and proper procedure remains extremely challenging and is demanded. Accordingly, we propose the simultaneous utilization of amphiphilic polythiophenes and QDs for TNP recognition. This approach can possibly integrate the feature of the light‐harvesting, low‐toxicity, and TNP‐binding properties of conjugated polythiophenes with the photostability and donor properties of QDs.

**Scheme 1:**
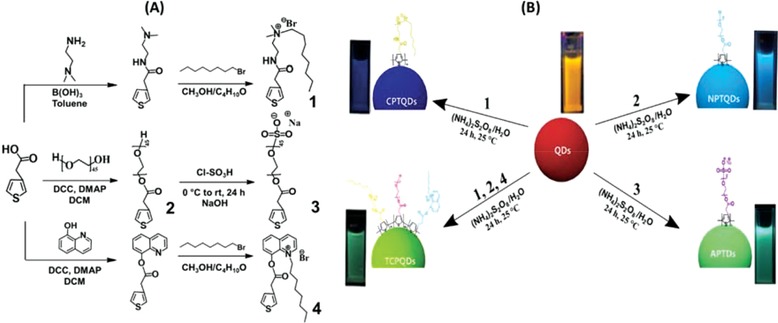
A) Reaction scheme for the synthesis of various amphiphilic thiophene monomers and B) synthesis of multicolor emissive amphiphilic conjugated polythiophene‐coated CdTe QDs via an in situ polymerization method.

In this study, highly sensitive and selective sensors based on novel nanohybrids, cationic polythiophene nanohybrids (CPTQDs), nonionic polythiophene nanohybrids (NPTQDs), anionic polythiophene nanohybrids (APTQDs), and thiophene copolymer nanohybrids (TCPQDs) for explosive‐TNP detection were (**Scheme** **1**). This method has five outstanding features: a) amphiphilic polythiophene ligands possess numerous advantages, namely, high quantum efficiency, low cytotoxicity, high sensitivity, and excellent photostability; b) this kind of ligands featured with hydrophobic π‐conjugated backbones and different hydrophilic groups (i.e., anionic, cationic, and nonionic) thereby endowing them with outstanding properties such as solubility in water, excellent photophysical properties patrimonial from their conjugated backbones, and considerable self‐assembly behavior due to their patrimonial amphiphilic structures; c) coating QDs with differently charged polythiophene ligands leads to formation of multicolor sensors with a different fluorescence emission and excitation ranges as well as enhancement of quantum yield (QY) for CPTQDs (75%) , NPTQDs (61%), APTQDs (72%), and TCPQDs (78%); d) combination between three different monomers can provide a remarkable fluorescence quenching based on inner filter effect (IFE) mechanism and contribution of electrostatic, π−π, and multiple hydrogen bonds interactions; e) integration of CdTe nanoparticles in polymer enhances the thermal stability, and decreases aggregation caused by π‐stacking.

Moreover, the potential application of this method was extended by utilizing the “PAD Analysis” application for smartphones on our designed paper‐based visual sensor and showed good results. Owing to the excellent fluorescent properties and nontoxicity of QD nanohybrids, the developed turn‐off sensors that can be applied for the rapid and selective sensing of TNP in tap and river water samples.

## 2 Results and Discussion

### 2.1 Highly Enhanced Fluorescence Intensity and Quantum Yield of CdTe QDs

**Figure 1:**
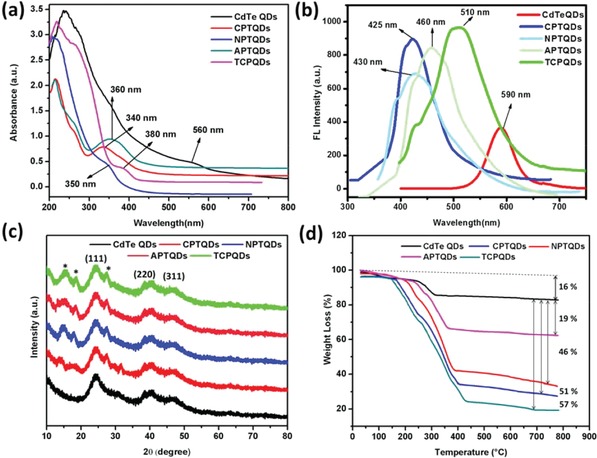
UV–vis absorption spectra a); PL spectra at different excitation wavelengths b); XRD patterns c); TGA analysis of CdTe QDs, CPTQDs, NPTQDs, APTQDs, and TCPQDs d).

**Figure** **1** shows the UV–vis and fluorescence spectra of the CdTe QDs and their nanohybrids. The UV–vis absorption of the CdTe QDs, CPTQDs, NPTQDs, APTQDs, and TCPQDs was detected at 560, 340, 350, 360, and 380 nm, respectively (Figure 1a). Compared to the noncoated CdTe QDs, the emission of QDs was considerably enhanced by coating with amphiphilic polythiophenes via in situ polymerization. During the formation of the conjugated polythiophenes shell, the emission of the CdTe QDs (590 nm) gradually decreased. Meanwhile, the CPTQDs, NPTQDs, APTQDs, and TCPQDs showed new peaks at ≈425, 430, 460, and 510 nm, respectively (Figure 1b), and the emission of these peaks enhanced steadily. Interestingly, compared to CdTe QDs, the fluorescence emission of the CPTQDs, NPTQDs, APTQDs, and TCPQDs was enhanced dramatically indicating 20, 10, 16, and 30 times stronger emission, respectively. The significant enhancement and the blue shifts in the emission intensity can be ascribed to the surface passivation of QDs because of coating with the polythiophenes shell. The QYs (Φ_x_) of CPTQDs, NPTQDs, APTQDs, and TCPQDs were determined to be 75%, 61%, 72%, and 78%, respectively, whereas the Φ_x_ of CdTe QDs was found to be only 5.6% (Table S1, Supporting Information) using rhodamine B as a reference. This remarkable enhancement of the QYs is probably due to the elimination of nonradiative decay pathways and surface passivation of CdTe QDs after coating with amphiphilic conjugated polythiophenes. The uncoated CdTe QDs with the polymers and the existence of surface states on the QDs result in a nonradioactive transition, which can reduce the radiative QY.^20^ However, when protected by conjugated polythiophenes that show a broad bandgap, the charge carriers are bounded in the core region and are extracted from the surface due to the great efficient offset of bandgap energies between the core and shell districts, which eventually generates dramatically enhanced QYs.^21^ This considerable enhancement in the photophysical properties indicates that these conjugated polythiophenes hold promise as shell materials.

### 2.2 Characterization of Amphiphilic Conjugated Polythiophene‐Coated CdTe QDs

The synthesized amphiphilic conjugated polythiophene‐coated CdTe QDs were characterized by FT‐IR (Figure S9, Supporting Information) and ^1^H‐NMR (Figures S10–S13, Supporting Information).

The XRD analysis of the QDs nanohybrids is shown in Figure 1c. The CdTe QDs showed diffraction peaks at 2θ values of 24.4, 40.4, and 47.2 that assigned to the 111, 220, and 311 planes of the zinc blend structure (JCPDF No. 75‐2086). The CPTQDs, NPTQDs, APTQDs, and TCPQDs exhibited the typical diffraction planes as the CdTe QDs in addition to polythiophenes peaks that appeared at 2θ values of 11.5, 17.0, and 26.0. Broadening of diffraction peaks indicates the formation of the nanosized CdTe QDs. Moreover, the observed decrease in the crystallinity of developed QDs may be explained by a small amount of amorphous Cd−thiolate complexes doped in the QDs.^22^

The thermal stability of the CdTe QDs and their nanohybrids were investigated by TGA (Figure 1d). In addition, the polymer amount on the QDs surface was determined using TGA analysis. A comparison of the TGA results of the CdTe QDs with those of the CPTQDs, NPTQDs, APTQDs, and TCPQDs revealed that the surface coatings of the CdTe QDs contained CPs to the amount of 51%, 46%, 19%, and 57%, respectively. These results revealed that the CdTe QDs were successfully coated using CPs.

**Figure 2:**
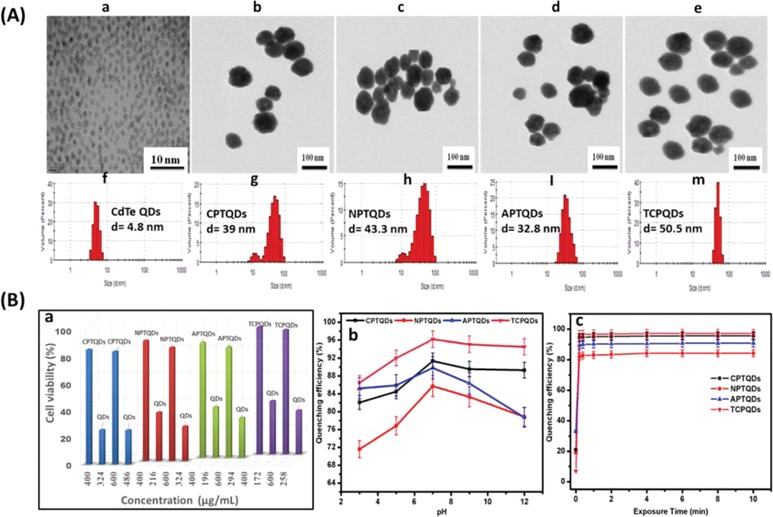
A) TEM images and size distribution using DLS of a,f) CdTe QDs, b,g) CPTQDs, c,h) NPTQDs d,I), and TCPQDs e, m) nanohybrids in phosphate buffer saline (PBS) solution (pH 7.4) and B) cell viability of HeLa cells in the presence of 400 and 600 µg mL^−1^ and their equivalent amount from bare QDs of the CdTe QDs, CPTQDs, NPTQDs, APTQDs, and TCPQDs a); effects of pH b) and incubation time c) on the fluorescence quenching of CPTQD, NPTQD, APTQD, and TCPQD sensors in the presence of TNP. The concentrations of TNP and the sensors are 5.0 and 10.0 × 10^−6^ m, respectively.

The morphological structures of the QD nanohybrids were investigated by TEM technique. **Figure** **2**A‐a shows that the CdTe QDs were dispersed effectively with the mean size of 5 nm. The CPTQDs, NPTQDs, APTQDs, and TCPQDs had similar morphology with an average size of approximately 39, 43, 31.5, and 49.5 nm, respectively, (Figure 2A‐b–e). The size distribution of nanoparticles was determined by DLS measurement. Figure 2A (f–m) shows that the hydrodynamic diameter of CdTe QDs, CPTQDs, NPTQDs, APTQDs, and TCPQDs was determined to be 4.8, 39, 43.3, 32.8, and 50.5 nm, respectively. These results revealed that the size of QDs was increased after the polythiophene coatings were applied. Both the TEM and DLS data were in good agreement with the size of the QD nanohybrids.

The ζ potential results of the QDs, CPTQDs, NPTQDs, APTQDs, and TCPQDs are summarized in Table S1 in the Supporting Information. The use of copolymers and nonionic polythiophenes (TCPQDs and NPTQDs) led to the formation of larger nanohybrids, 50.5 and 43.3 nm, respectively, whereas the anionic and cationic polythiophenes produce nanohybrids of 32.8 and 39.0 nm, respectively.

Energy dispersive X‐ray spectroscopy (EDX) measurement was performed to emphasize the presence of accurate elements in the corresponding prepared nanohybrids, as demonstrated in Figure S14 in the Supporting Information. The appearance of Cd and Te suggests the presence of CdTe in the CPTQDs, NPTQDs, APTQDs, and TCPQDs. The elemental compositions of all nanohybrids are provided in Table S2 in the Supporting Information.

To confirm the biocompatibility of QDs coated with amphiphilic polythiophenes (CPTQDs, NPTQDs, APTQDs, and TCPQDs) compared with bare QDs, MTT test (3‐(4,5‐dimethylthiazol‐2‐yl)‐2,5‐diphenyltetrazoliumbromide) against HeLa cells was conducted. The equivalent content of QDs in QDs coated with amphiphilic polythiophenes was calculated via thermogravimetric analysis (TGA) results (Figure 1d), which enables the determination of the polymers amount on the QDs surface. The surface‐coated QDs showed low cytotoxicity at the concentrations of 600 and 400 µg mL^−1^ to the HeLa cells. On the contrary, equivalent content of bare QDs (Table S3, Supporting Information) was toxic to the HeLa cells showing decreased cell viability in a concentration‐dependent manner as illustrated in Figure 2B‐a. The surface‐coated QDs demonstrated lower cytotoxicity due to the shell of conjugated polythiophenes, which effectively prevents the cadmium ions to interact with cells.^23^ Our results are consistent with those in the literature, where the reducing of QDs cytotoxicity by the surface coating with amphiphilic polymers has been reported.^24^ These results indicate that the developed materials could be a great candidate as the optical sensors in practice.

### 2.3 Optimization of the Experimental Conditions

The conditions under which the fluorescence properties of nanohybrid sensors were optimized were mainly pH and response time. Figure 2B‐b demonstrates the effects of pH (3.0–12.0) on the fluorescence quenching efficiency of the CPTQDs, NPTQDs, APTQDs, and TCPQDs sensor in the presence of 5.0 × 10^−6^ m TNP. As shown in Figure 2B‐b, the quenching ability of TNP toward the CPTQDs, NPTQDs, APTQDs, and TCPQDs reached its maximum at pH 7.0, which is highly likely owing to the strong ability of the sensor sites to bind with TNP. This result has rendered the sensors suitable for application to real water samples because environmental water samples are usually neutral. Therefore, the next experiments were performed at pH 7.0.

The detection response time for TNP using the proposed sensors were performed and the quenching efficiency versus incubation time is plotted in Figure 2B‐c, which illustrates a rapid response time for TNP detection. For example, the addition of TNP (5.0 × 10^−6^ m) caused fluorescence quenching of the CPTQDs, NPTQDs, APTQDs, and TCPQDs to the effect of 91%, 85%, 89%, and 96%, respectively, within 60 s of incubation time. Thus, QD nanohybrids can be considered as candidate sensors for the rapid determination of TNP.

### 2.4 Sensitivity of TNP Detection

**Figure 3:**
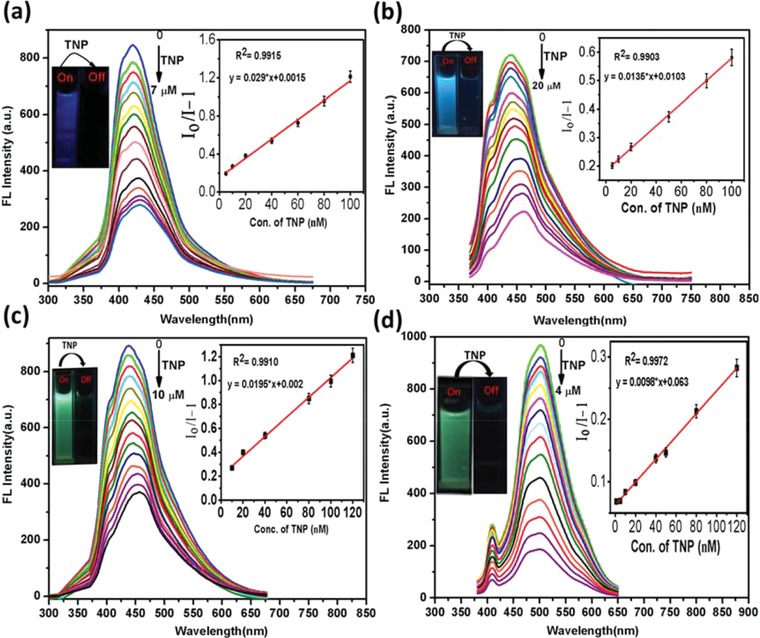
Fluorescence emission spectra of CPTQDs a), NPTQDs b), APTQDs c), TCPQDs d) in PBS buffer solution (pH 7.0) containing different concentrations of TNP (0–7, 0–20, 0–10, and 0–4 × 10^−6^ m, respectively). The insets (right) show the *I*_o_/*I* − 1 plotted against the TNP concentration (×10^−9^ m). The insets (left) show the color of the sensor solutions in PBS before and after adding TNP (irradiation under UV lamp at 365 nm).

Under the optimized conditions, the efficiency of the CPTQDs, NPTQDs, APTQDs, and TCPQDs sensor to quantitatively determine TNP was further evaluated. **Figures** **3**a–d shows that the increase in the amount of TNP causes the fluorescence intensities to gradually decrease. Furthermore, when high amount of TNP are added to solutions of the CPTQDs, NPTQDs, APTQDs, and TCPQDs, the emission peak was observed to undergo red shift, indicating the existence of electron transfer between TNP and the sensors.^25^ As depicted in the inset of Figure 3a–d) the value *I*_o_/*I* − 1 shows a linear relationship for TNP in the ranges from 5–100, 10–100, 10–120, and 1–120 × 10^−9^ m with correlation coefficients of 0.9915, 0.9903, 0.9910, and 0.9912 for the CPTQDs, NPTQDs, APTQDs, and TCPQDs, respectively. The limits of detection, LODs = 3σ/*s*, where “σ” and “*s*” are the standard deviation of the corrected blank signals of the QD nanohybrids and the slope of the calibration curve, respectively, which were calculated to be 2.56, 7.23, 4.12, and 0.56 × 10^−9^ m for the CPTQDs, NPTQDs, APTQDs, and TCPQDs, respectively. It should be noted that the LOD values of the proposed sensors are much lower than other reported (Table S4, Supporting Information) and this level of sensitivity occurs below the maximum permissible concentration of TNP in drinking water (0.25 × 10^−6^ m) as established by the US.^26^ Moreover, under 365 nm UV light, the quenching of sensors can be seen visually (inset of Figure 3a–d) in which the color of the sensors visibly darkened as soon as TNP was added.

### 2.5 Selective Detection of TNP Explosive

**Figure 4:**
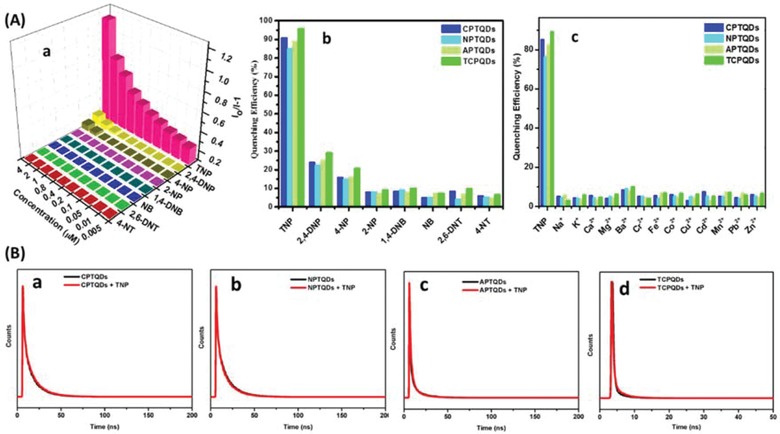
A) S–V plots of TCPQDs a); selectivity study of CPTQD, NPTQD, APTQD, and TCPQD sensors in the presence of other nitroaromatics b); selectivity study of CPTQDs, NPTQDs, APTQDs and TCPQDs sensors in the presence of metal ions c) and B) time‐resolved decay of the CPTQDs a), NPTQDs b), APTQDs c), and TCPQDs d) in the absence and presence of TNP in 10 × 10^−3^ m PBS (pH 7.0).

The designed CPs are rich with electron‐donating groups and the integration of QDs increases the electron density of the polymers. Thus, we assumed the effective interaction between the electron‐rich sensors and electron‐deficient TNP. **Figure** **4**A‐a and Figure S15 in the Supporting Information illustrate the fluorescence quenching response of the developed sensors to various explosives including 4‐nitrotoluene (4‐NT), 2,6‐dinitrotoluene (2,6‐DNT), nitrobenzene (NB), 1,4‐dinitrobenzene (1,4‐DNB), 2‐nitrophenol (2‐NP), 4‐nitrophenol (4‐NP), 2,4‐dinitrophenol (2,4‐DNP), and TNP. The results show that the fluorescence intensities of the sensors were strongly quenched by TNP. The thiophene copolymer nanohybrids with two different cationic monomers (TCPQDs) and CPTQDs produced the highest quenching response, whereas the response obtained from the APTQDs and NPTQDs was less marked. The positively charged TCPQD and CPTQD surfaces facilitate binding with the —OH group of TNP, and subsequently promote more efficient quenching. Moreover, the presence of negative charge on the surface of APTQDs raises the electron density of the polymer, resulting in boosted interaction between the APTQDs and TNP. In contrast, the neutral charge on the surface of the NPTQDs reduces the electron density and the interaction of the NPTQDs with TNP. Furthermore, Figure 4A‐a and Figure S15 in the Supporting Information clearly show that TNP causes more fluorescence quenching efficiencies than other analogs. The quenching efficiency was estimated by determining the quenching constant (*K*_S‐V_) using the Stern–Volmer (S–V) Equation (1)

(1) $$I_{0}/I=K_{\mathrm{S–V}}\left[Q\right]+1$$

where *I*_0_ and *I* are the fluorescence intensities before and after the addition of analytes, [*Q*] is the concentration of analytes, and *K*_S−V_ is the quenching constant (m ^−1^). The *K*_S−V_ and *R* ^2^ parameters for all analytes were calculated using Equation (1) and given in **Table** **1**. Figure 4A‐a and Figure S15 in the Supporting Information displayed that TNP shows an exponential S–V curve, whereas others show a linear curve. The S–V plots of TNP are close to linear at lower concentration, but they deviate from linearity and increase exponentially at higher concentration. For other nitroexplosives, the S–V plots remain linear at higher concentration. TNP possesses the highest *K*_S−V_ (1.41, 0.99, 1.22, and 1.91 × 10^5^ m ^−1^, for the CPTQDs, NPTQDs, APTQDs, and TCPQDs, respectively), which is quite larger than those of other nitroexplosives (Table 1). These results indicate superior selectivity of the proposed sensors for TNP detection compared to other nitroaromatics.

**Table 1:** The parameters for each quencher related to the fitted Stern−Volmer model

Analytes	CPTQDs		NPTQDs		APTQDs		TCPQDs	
	*K*_S‐V_ × 10^3^	*R* ^2^	*K*_S‐V_ × 10^3^	*R* ^2^	*K*_S‐V_ × 10^3^	*R* ^2^	*K*_S‐V_ × 10^3^	*R* ^2^
TNP	141	0.9927	99	0.9982	122	0.9760	191	0.997
2,4‐DNP	2.26	0.9823	1.28	0.9813	2.20	0.9755	5.00	0.987
4‐NP	1.25	0.9845	1.21	0.9849	1.15	0.9785	5.20	0.996
2‐NP	0491	0.9867	0.49	0.9886	0.491	0.9867	2.00	0.976
1,4‐DNB	0.219	0.9553	0.21	0.9534	0.219	0.9353	0.47	0.989
NB	0.142	0.9579	0.30	0.9575	0.341	0.9575	0.59	0.985
2,6‐DNT	0.104	0.9845	0.14	0.9845	0.144	0.9845	0.33	0.982
4‐NT	0.132	0.9819	0.13	0.9818	0.132	0.9818	0.30	0.980

As presented in Figure 4A‐b and Figure S16 in the Supporting Information, the 2,4,6‐TNP, 2,4‐DNP, 4‐NP, and 2‐NP showed higher quenching effect than other nitroaromatics. Figure S16 in the Supporting Information clearly reveals that the quenching efficiency decreases with the decrease in acidity of nitrophenols compound (the pKa values of 2,4,6‐TNP, 2,4‐DNP, and 4‐NP are 0.38, 4.11, 7.15, and 7.23, respectively). Since TNP is a stronger acid compared to 2,4‐DNP, 4‐NP and 2‐NP, it more tends to interact with CPTQDs, NPTQDs, APTQDs, and TCPQDs via acid−base interaction to form a stable electrostatic complex.[[qv: 25a,27]] The quenching efficiencies by these analytes follow the order 2,4,6‐TNP > 2,4‐DNP > 4‐NP > 2‐NP. In addition, the CPTQDs, NPTQDs, APTQDs, and TCPQDs also exhibited highly sensitive detection of TNP with high quenching efficiency (91%, 85%, 89%, and 96%, respectively). In spite of the variation of acidic environments, the TCPQDs also exhibited absolute fluorescence quenching toward TNP, compared with other phenolic explosives (Figure S17a, Supporting Information). The best selectivity was obtained for the TCPQDs, which were clearly observed to selectively sense only TNP. Furthermore, the selectivity of the sensors was investigated in the existence of Na^+^, Li^+^, Ca^2+^, Ba^2+^ Cr^2+^, Fe^2+^, Co^2+^, Cu^2+^, Cd^2+^, Mn^2+^, Pb^2+^, and Zn^2+^ ions at a concentration of 1 × 10^−3^ m. As a result, the quenching efficiency of TNP is shown to be superior to that of all these metal ions (Figure 4A‐c), suggesting the great selectivity of the TNP sensors. For comparison, the emission of bare QDs (capped with mercaptoacetic acid) could be quenched by Cu^2+^ and Fe^2+^ (Figure S17b, Supporting Information). This quenching effect was attributed to the adsorption of the metal ions on the trap sites of the QD surface and concomitant formation of CuS or FeS particles, which eventually result in the surface passivation of the QDs. Also, it was reported that the metal ions nonspecifically bound to the QD surface that facilitates the nonradiative electron/hole recombination.^28^ On the other hand, the metal ions showed no interference to the modified CdTe QDs that may be due to the lack of proper binding sites for metal ions. Moreover, the coating of QDs with CPs would result in a thicker ligands shell (QDs size before coating is 4.8 nm and after coating ranges from 32.8–50.5 nm, see Figure 2A) preventing the access of Cu^2+^ and Fe^2+^ to QDs surface and avoid the quenching effect of metal ions.^29^

### 2.6 Mechanism of TNP Detection

**Figure 5:**
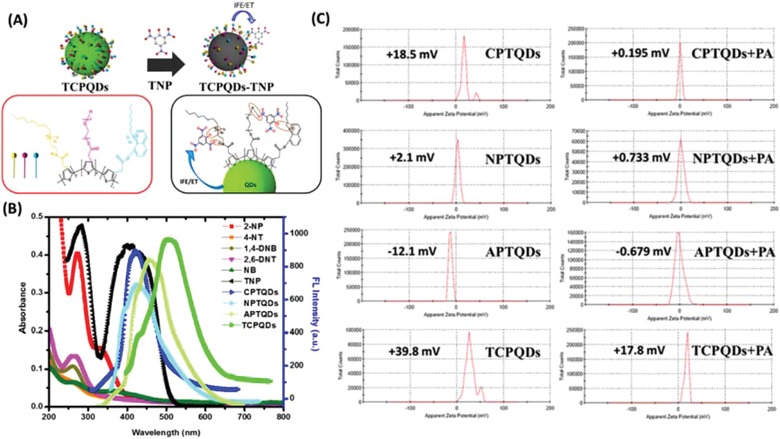
A) Schematic representation of the use of the TCPQD sensor to detect TNP via IFE and a molecular interaction mechanism; B) spectral overlap between emission spectra of CPTQDs, NPTQDs, APTQDs, and TCPQDs and absorption spectra of different nitroaromatic explosives; and C) zeta potential distribution plots of the CPTQDs, NPTQDs, APTQDs and TCPQDs in the absence and presence of TNP in PBS buffer solution (pH 7.0).

The possible mechanism whereby TNP is detected by the proposed sensors is supposed to be Förster resonance energy transfer (FRET) or IFE, and molecular interactions through electrostatic, π–π, and hydrogen bonding between QDs coated with conjugated polythiophenes and TNP. In the case of FRET or IFE processes, considerable spectral overlap occurs between the absorption spectrum of an analyte and the emission spectrum of the sensor.^30^ A time‐resolved fluorescence test was conducted to confirm the nature of quenching. Figure 4B shows the decay time of the CPTQDs, NPTQDs, APTQDs, and TCPQDs before and after addition of TNP using pulse excitation at 375 nm. The lifetime of the QD nanohybrids remained constant after the addition of a definite concentration of TNP, namely, 7 × 10^−6^ m for the CPTQDs, 20 × 10^−6^ m for the NPTQDs, 10 × 10^−6^ m for the APTQDs, and 7 × 10^−6^ m for the TCPQDs. The lifetimes in the absence and presence of TNP are 18.88 and 18.90 ns for the CPTQDs, 16.39 and 16.44 ns for the NPTQDs, 18.61 and 18.59 ns for the APTQDs, and 20.01 and 20.19 ns for the TCPQDs, respectively. These results suggest the presence of static quenching via the formation of ground state electrostatic interactions and exclude the possibility of excited state energy transfer via the FRET process. Therefore, the IFE could be considered as one process in the fluorescence quenching. Furthermore, the selectivity toward TNP could also be explained by the IFE mechanism. The absorption spectrum of TNP highly overlaps with the emission spectra of the CPTQDs, NPTQDs, APTQDs, and TCPQDs, resulting in strong quenching in fluorescence intensities compared to other nitrophenols (2,4‐DNP, 4‐NP, and 2‐NP) or other nitroaromatics that have small overlap as displayed in **Figure** **5**B and Figure S18a–b in the Supporting Information, which resulted in poor IFE efficiency. Moreover, the fluorescence quenching could be caused by molecular interactions such as electrostatic and hydrogen bonding interactions between the ‐OH group on the benzene ring of electron‐deficient TNP and the free basic sites ((‐+NR3, ‐NH‐, ‐OH, and C=O on the surface) of the electron‐rich QD nanohybrids, and π‐π interaction between their benzene and thiophene rings. To confirm the role of electrostatic interaction in the fluorescence detection process, the fluorescence characteristics of some nitroaromatic explosives containing one hydroxyl group, such as DNP and NP, were investigated. The quenching efficiency decreased in the order TNP > DNP > NP, which is in accordance with their order of acidity (Figure S18b, Supporting Information). Since, TNP consists of three electron‐deficient —NO_2_ groups that boost its ability to dissociate in aqueous medium, a more favorable electrostatic interaction between TNP and QDs is expected compared to the 2,4‐DNP or 4‐NP, which subsequently enhanced the quenching efficiency. In spite of the variation of acidic environments, the TCPQDs also exhibited absolute fluorescence quenching toward TNP compared with other phenolic explosives (Figure S17a, Supporting Information) indicating that acid itself cannot quench the fluorescence of QDs and molecular interactions is the main factor behind the quenching process. The molecular interactions can also be well‐verified by the red shift in the emission peak upon the addition of high concentrations of TNP to the QD nanohybrids.[[qv: 24a]] Thus, compared to other nitroaromatic explosives, the QD nanohybrids exhibited much higher fluorescence quenching response toward TNP because of a favorable molecular interaction, including electrostatic, hydrogen bonding, and π–π interactions. Based on above discussion, we can clearly disclose that the highly efficient fluorescence quenching of TNP on QDs comes from molecular interactions, including electrostatic, hydrogen bonding, and π–π interactions‐assisted IFE (Figure 5A and Scheme S2, Supporting Information).

To confirm the existence of molecular interactions (hydrogen bonds and electrostatic interaction) between the CPTQDs, NPTQDs, APTQDs, and TCPQDs and TNP the FT‐IR and zeta‐potential (ζ) analyses were carried out. The FT‐IR spectra of the CPTQDs, NPTQDs, and APTQDs are presented in Figure S19a–c in the Supporting Information. The N—H, O—H, and C=O bonds were observed at 1570, 3450, and 1737 cm^−1^, which are then notably shifted to 1520, 3370, and 1717 cm^−1^, respectively, after interaction with TNP. Meanwhile, the O—H and N—H bonds on the surface of the TCPQDs (Figure S19d, Supporting Information) were observed at 3500 and 1580 cm^−1^, which are also shifted considerably to 3420 and 1561 cm^−1^, respectively, after addition of TNP.^31^ The electrostatic interaction was also confirmed by obtaining the variation presented in Figure 5C. Initially, the CPTQDs, NPTQDs, APTQDs, and TCPQDs showed ζ values of approximately +18.5, +2.1, −12.1, and +39.8 mV, respectively, changing to approximately +0.195, +0.733, −0.679, and +17.8 mV after interaction with TNP.^32^ This remarkable variation in ζ values of the sensors confirms the electrostatic interaction between the sensors and TNP.

### 2.7 Practical Application of the Sensor to Environmental Water Samples

The practical applicability of the new sensors was investigated by conducting experiments to detect TNP in tap and river water samples diluted 50‐fold and spiked with different concentrations of TNP (0.04, 0.80, and 1.60 × 10^−6^ m). As shown in **Table** **2**, good recoveries of TNP using the CPTQDs, NPTQDs, APTQDs, and TCPQDs were reached to recover 100.06%, 98.02%, 106.75%, and 106.37%, with relative standard deviations (RSDs) of 1.88%, 3.72%, 2.14%, and 2.32% in tap water samples. Meanwhile, the recoveries for the spiked river water samples were 105.13%, 107.50%, 103.02%, and 101.47% with RSDs of 3.12%, 3.11%, 2.01%, and 3.44%, respectively. The results indicated the feasibility of this method for the accurate detection of traces of TNP in environmental samples, demonstrating its great potential for practical applications.

**Table 2:** Detection of TNP in real samples using the amphiphilic conjugated polythiophene‐coated CdTe QDs

Sensors	Tap water				River water			
	Spiked [×10^−6^ m]	Found [×10^−6^ m]	Recovery [%]	RSD % (*n* = 5)	Spiked [×10^−6^ m]	Found [× 10^−6^ m]	Recovery [%]	RSD % (*n* = 5)
CPTQDs	0.04	0.037	92.50	3.66	0.04	0.038	97.50	2.34
	0.80	0.796	98.49	2.34	0.80	0.737	92.15	1.77
	1.60	1.601	100.06	1.88	1.60	1.682	105.13	3.12
NPTQDs	0.04	0.038	95.00	2.01	0.04	0.043	107.50	3.11
	0.80	0.760	95.02	2.22	0.80	0.781	97.57	2.08
	1.60	1.568	98.02	3.72	1.60	1.496	93.500	2.23
APTQDs	0.04	0.041	102.50	3.37	0.04	0.040	100.00	2.24
	0.80	0.854	106.75	2.14	0.80	0.824	103.02	2.01
	1.60	1.515	94.69	2.76	1.60	1.542	96.38	1.89
TCPQDs	0.04	0.041	102.50	2.11	0.04	0.038	95.00	2.81
	0.80	0.687	85.87	1.54	0.80	0.812	101.47	3.44
	1.60	1.705	106.37	2.32	1.60	1.587	99.19	2.92

### 2.8 Visual Detection of TNP Using Paper Strips and Fluorescent Chitosan Film‐Based Sensors

**Figure 6:**
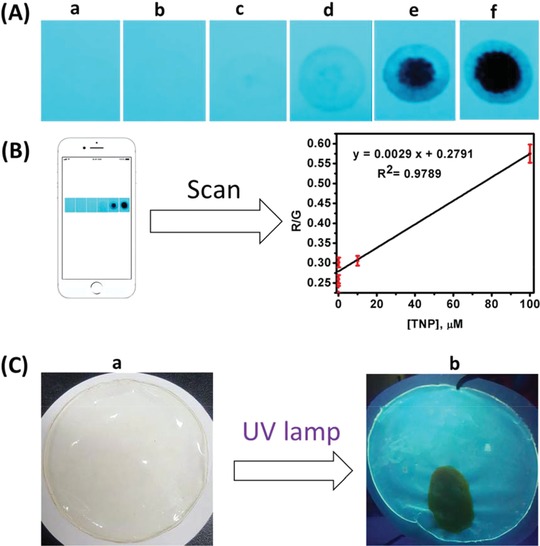
A) Color of fluorescent paper test strips under UV light before and after adding 10 µL of various concentrations of TNP solution: drop of water a), 10^−12^ b), 10^−9^ c), 10^−7^d), 10^−5^ e), 10^−4^ M f); B) calibration plot using our visual sensor based on paper strips in combination with a smartphone application for TNP detection; C) TCPQDs‐doped transparent chitosan film in daylight a), and visualization of TCPQDs‐doped chitosan fluorescent film under UV light with dark spot resulting from left thumb impression with TNP residual (lamp excitation at 365 nm) b).

A coating of the TCPQD nanohybrid was applied to the surface of paper strips, which were then dipped into TNP solutions with various concentrations (1 × 10^−4^ to 1 × 10^−12^ m). The emission of the TCPQD sensor was quenched right after adding different concentrations of TNP that could be observed under a 365 nm UV lamp (**Figure** **6**A). The paper strips can detect concentration down to 1 × 10^−9^ m (2.29 pg) from TNP which can be also tracked by the naked eyes. To develop a portable paper‐based sensor, images were captured with a smartphone and then scanned with the “PAD Analysis” application.^33^ The difference in the red (R) and green (G) color intensities of the images depicting various concentrations of TNP was obtained. Using the R/G ratio and concentration of TNP, the curve can be described by the equation: *y* = 0.0029*x* + 0.2791 (*R* ^2^ = 0.9789); where “*y*” is the R/G and “*x*” is the concentration of TNP (Figure 6B). This result demonstrates the ability of the TCPQD‐coated paper‐based visual sensor to adapt to the smartphone APP for the on‐site quantitative detection of TNP.

A fluorescent film was also prepared by doping commercial chitosan (CS) with 5% TCPQDs (Figure 6C‐a) and was used to describe its fluorescence response towards TNP. A thumb was rubbed with TNP and then the finger was pressed against the fluorescent film. The fluorescence quenching in the zone covered by the fingerprint could be observed when illuminated with UV light (Figure 6C‐b). This result indicated that the detection of TNP in the solid state could also be achieved by the TCPQDs‐CS film.

### 2.9 Method Performance Comparison

The sensitivity of the developed sensors for the detection of TNP was compared with bare QDs (Figure S20, Supporting Information). As shown in Figure S15 in the Supporting Information, no significant changes were observed at the fluorescence intensity of bare CdTe QDs by adding TNP, whereas the fluorescence intensity of QDs‐modified polymer was strongly quenched by TNP. The sensitivity of our method was compared also with other reported ligands‐modified QDs as listed in Table S4 in the Supporting Information. It can be seen that although some methods are very sensitive, but they suffer from such limitations as low selectivity,^34^ long analysis time,^35^ complicated micelles system,^36^ using toxic organic solvent, or the detection not completely in aqueous media.^37^ It is noteworthy that the LODs in this work are much lower than that of reported methods.^36^, ^38^ Our developed sensors system does not require any complex procedures or expensive instruments, and demonstrates ultrasensitivity and selectivity as well as fast detection to TNP (60 s). The amphiphilic polythiophenes structure played a key role in higher sensitivity compared to abovementioned methods. The ultrasensitivity observed with the proposed sensors that is consistent with the enhanced molecular interactions properties resulting from the electrostatic, π−π and multiple hydrogen bonding interactions. These interactions lead to the improved binding to TNP and to the more efficient quenching of the sensors. Furthermore, the ultrasensitivity of these sensors for TNP likely due to their higher fluorescence QYs (78% for TCPQDs, 75% for CPTQDs, 61% for NPTQDs, and 75% APTQDs).

## 3 Conclusion

In conclusion, highly sensitive and selective sensors based on novel nanohybrids, CPTQDs, NPTQDs, APTQDs, and TCPQDs for explosive‐TNP detection were developed. The fluorescence of sensors was selectively quenched by the addition of TNP due to the IFE and molecular interaction mechanisms. The LOD for TNP was determined to be 0.56 × 10^−9^ m using TCPQDs sensor, which is exceptionally low. We also applied these sensors in tap and river water samples and showed remarkable sensitivity with acceptable recovery results. Furthermore, a facile paper sensor for TNP detection was developed successfully using filter paper coated with TCPQDs, thereby enabling us to detect TNP by naked eyes. More interestingly, this paper sensor was coupled with a smartphone to make it suitable for on‐site application. We also designed TCPQD‐doped chitosan film to visualize the quantitative detection of TNP. The developed method proposes reliable and accurate detection of TNP, which is foreseen to open new avenues for the development of facile sensing strategies to prevent environmental contamination and terrorist threats.

## 4 Experimental Section

All materials, instrumentation, and methods are provided as the Supporting Information.

## Conflict of Interest

The authors declare no conflict of interest.

## Supporting information

Supplementary
