# Supplementary material for: Multiple Emitting Amphiphilic Conjugated Polythiophenes‐Coated CdTe QDs for Picogram Detection of Trinitrophenol Explosive and Application Using Chitosan Film and Paper‐Based Sensor Coupled with Smartphone
Source: Adv Sci (Weinh). 2018 Nov 12;6(2):1801467. doi: 10.1002/advs.201801467 (PMC6343090; doi:10.1002/advs.201801467)
Supplement: Supplementary file 1 — Supplementary [file ADVS-6-1801467-s001.pdf]

Copyright WILEY-VCH Verlag GmbH & Co. KGaA, 69469 Weinheim, Germany, 2018.

ADVANCED SCIENCE
Open Access

# Supporting Information

for *Adv. Sci.,* DOI: 10.1002/advs.201801467

# Multiple Emitting Amphiphilic Conjugated Polythiophenes-Coated CdTe QDs for Picogram Detection of Trinitrophenol Explosive and Application Using Chitosan Film and Paper-Based Sensor Coupled with Smartphone

*Salah M. Tawfik, Mirkomil Sharipov, Sarvar Kakhkhorov, Mohamed R. Elmasry, and Yong-Ill Lee**

# Supporting Information

## Multiple Emitting Amphiphilic Conjugated Polythiophenes-Coated CdTe QDs for Picogram Detection of Trinitrophenol Explosive and Application Using Chitosan Film and Paper-Based Sensor Coupled with Smartphone

Salah M. Tawfik [1,2], Mirkomil Sharipov[1], Sarvar Kakhkhorov[1], Mohamed R. Elmasry[1], Yong-Ill Lee [1,*]

[1] Anastro Laboratory, Department of Chemistry, Changwon National University, Changwon 51140, Republic of Korea

[2]Egyptian Petroleum Research Institute (EPRI), Nasr City, Cairo11727, Egypt

* Corresponding author
E-mail: yilee@changwon.ac.kr (Y-I Lee)
Tel./Fax. (+82) 55-213-3436/213-3439

# 1. Experimental section

**Caution!** Nitroaromatic analytes are classified as secondary chemical explosives and should be handled only in small quantities.

## 1. 1. Materials and methods

All nitroaromatics were obtained from Sigma-Aldrich (Scheme S1) and used very carefully in the experiments. The 3-thiopheneacetic acid (98%), N,N-dimethylethylenediamine (95%), 1-bromooctadecane (97%), 8-hydroxyquinoline, chlorosulfonic acid, boric acid (99.8%), dicyclohexylcarbodiimide (DCC, 99%), 4-(N,N-dimethylamino)-pyridine (DMAP, 98%), N-hydroxysuccinimide (NHS, 98%), polyethylene glycol ($M_n$ 2000), and chitosan were also obtained from Sigma-Aldrich and used without further purification. The distilled water used in all experiments had a resistivity higher than 18 $M\Omega \cdot cm^{-1}$ from a Milli-Q water purification system. Whatman® grade 1 filter paper (10.0 cm) was used as paper strips.

## 1.2. Instrumentation

FT-IR spectra were recorded on an FT/IR-6300 Fourier Transform Infrared Spectrometer (Jasco, Japan). $^1$H-NMR spectra were recorded at 400 MHz using a Bruker NMR instrument. The fluorescence experiments were performed on an FP-6500 spectrofluorometer (Jasco, Japan) using a quartz cuvette with a 1-cm path length. The absorption spectra were obtained using an Agilent 8543 (Agilent, USA) UV/Vis spectrophotometer. TEM measurements were carried out using a JEM-2100F transmission electron microscope (JEOL, Tokyo, Japan) operating at 200 kV. The XRD powder pattern was obtained on an X'Pert PRO MPD X-ray diffractometer (Analytical, Netherlands) with Cu Kα (ratio $K_{\alpha2}/K_{\alpha1}$=0.5) radiation. Thermogravimetric analysis (TGA) was carried out using an SDT Q600 V20.9 Build 20 instrument at a heating rate of 10 °C/min with a constant $N_2$ flow rate of 20 mL/min within the temperature range of 35-800 °C. DLS analysis was carried out using a Zetasizer Nano ZS90 apparatus (Malvern Instruments, Worcestershire, U.K.). The time-resolved photoluminescence study was carried out using an Edinburgh Instruments FL920 fluorescence lifetime spectrometer with a laser excitation source of 375 nm.

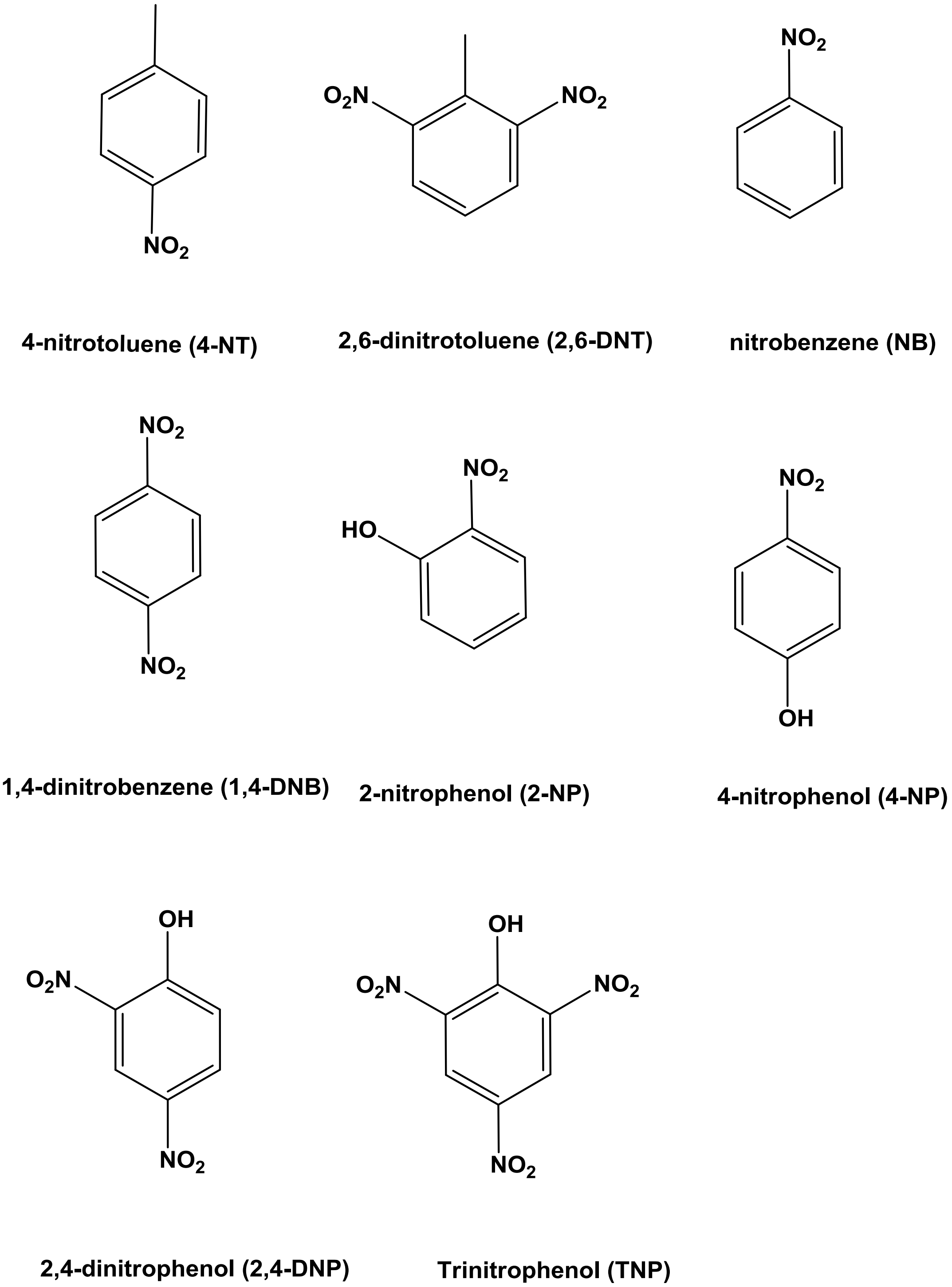


**Scheme S1. Structure of the experimental nitroaromatics**

## 1.2. Synthesis

### 1.2.1. Synthesis of N-(2-(dimethylamino)ethyl)-2-(thiophen-3-yl)acetamide

Boric acid (0.031 g, 0.5 mmol) was added to a solution of 3-thiophene acetic acid (0.711 g, 5.0 mmol) in toluene (100 mL). N,N-dimethylethylenediamine (0.443g, 5.0 mmol) was then

added in one portion. The reaction mixture was refluxed for 8 h and water was collected azeotropically in the Dean–Stark trap. The mixture was allowed to cool to 40–45 °C, filtered to remove the boric acid present in the reaction mass and further cooled to 25–35 °C. After stirring for 1 h at 25–35 °C, toluene was decanted, and then the resulting crude material was dissolved in methanol (50 mL). Distillation afforded the product (1.01 g, yield 94.92%) as syrup. FT-IR (KBr, Figure S1), υ= 3280 $cm^{-1}$ (N–H), 3070 $cm^{-1}$ (=C–H), 2910 $cm^{-1}$ (C–H), 1640 $cm^{-1}$ (C=O amide), 1520 $cm^{-1}$ (N-H bond), 1450 $cm^{-1}$ (C=C), 1125 $cm^{-1}$ (C–N). 750 $cm^{-1}$ (C–S). $^{1}$H-NMR ($D_2O$, 400 MHz, Figure S2): δ= 7.98 (t, 1H, N-H), 7.46 (s 1H, thiophene moiety), 7.22 (d, 1H, thiophene moiety), 7.02 (d, 1H, thiophene moiety), 3.45 (s,2H, $-CH_2$), 3.19 (m, 2H, $-CH_2$), 2.28 (m, 2H, $-CH_2$), 2.106 (s, 6H, $-CH_3$).

**1.2.2. Synthesis of N, N-dimethyl-N-(2-(2-(thiophen-3-yl)acetamido)ethyl)octan-1-aminium bromide (monomer 1)**

1-Bromooctane (0.193 g, 1 mmol) was dissolved in 20 mL of $CH_3OH/(C_2H_5)_2O$ (v/v = 3/2) with the subsequent addition of N-(2-(dimethylamino)ethyl)-2-(thiophen-3-yl)acetamide (0.276 g, 1.3 mmol). The mixture was stirred at room temperature for 12 h. After the reaction was completed, the reaction solution was concentrated to 5 mL. The residue was poured into 200 mL of absolute diethyl ether under stirring and then filtered. The precipitate was filtered, washed with absolute diethyl ether and dried to give yellow waxy compound **monomer 1** (0.44 g, yield 93.89%). FT-IR (KBr, Figure S1), υ= 3305 $cm^{-1}$ (N–H), 3075 $cm^{-1}$ (=C–H), 2950 $cm^{-1}$ (C–H asy), 2840 $cm^{-1}$ (C–H sy), 2680 $cm^{-1}$ ($C–N^{+}$), 1650 $cm^{-1}$ (C=O amide), 1533 $cm^{-1}$ (N-H bond), 1470 $cm^{-1}$ (C=C), 1350 $cm^{-1}$ ($(CH_2)_n$), 1150 $cm^{-1}$ (C–N). 755 $cm^{-1}$ (C–S). $^{1}$H-NMR ($D_2O$, 400 MHz, Figure S2): δ= 7.95 (t, 1H, N-H), 7.28 (s, 1H, thiophene moiety), 7.15 (d, 1H, thiophene moiety), 6.95 (d, 1H, thiophene moiety), 3.55 (s, 2H, $-CH_2$), 3.41 (m, 2H, $-CH_2$), 3.11 (m, 2H, $-CH_2$), 2.95 (s, 6H, $-CH_3$), 2.75-1.11(m, 14H, alkyl chain), 0.88 $cm^{-1}$ (t, 3H, $-CH_3$).

**1.2.3. Synthesis of monomer 2**

3-thiopheneacetic acid (0.142 g, 1 mmol), PEG-2000 (10 g, 5 mmol), DCC (0.206 g, 1 mmol) and DMAP (0.0244 g, 0.2 mmol) were dissolved in anhydrous DCM (60 mL) and stirred at room temperature for 24 h. The mixture was centrifuged, and the supernatant was concentrated in vacuo (20 mbar, 30 °C), redissolved in DCM (10 mL), and washed with 1 mM HCl (pH 3) (3 × 30 mL), saturated $NaHCO_3$ (3 × 30 mL), and $H_2O$ (3 × 30 mL). The

organic phase was dried over $MgSO_4$ for 12 h, filtered, and concentrated in vacuo (20 mbar, 30 °C) to give monomer **2** as a white solid (1.95 g, yield 91.80 %). FT-IR (KBr, Figure S3), υ= 3470 $cm^{-1}$ (O–H), 3100 $cm^{-1}$ (=C–H), 2900 $cm^{-1}$ (C–H), 1736 $cm^{-1}$ (C=O ester), 1480 $cm^{-1}$ (C=C), 1125 $cm^{-1}$ (C–O), 839 $cm^{-1}$ (C–S). $^1$H-NMR ($D_2O$, 400 MHz, Figure S4): δ= 7.38 (s, 1H, thiophene moiety), 7.23 (d, 1H, thiophene moiety), 7.01 (d, 1H, thiophene moiety), 3.55 (s, 2H, -$CH_2$), 3.65-4.22 (m, 4H, $OCH_2CH_2$ in PEG unit), 2.05 (t, 1H, -OH).

### 1.2.4. Synthesis of monomer 3

At 0 °C, chlorosulfonic acid (0.583 g, 5 mmol) was added to a solution of monomer 2 (2.124 g, 1 mmol) in dichloromethane (20 mL), and the resulting solution was stirred at room temperature overnight. Then, the solution was concentrated under vacuum, and ether was added to it. The resulting precipitate was filtered and washed with ether three times to get THP-PEG-$OSO_3H$ as a gummy solid. THP-PEG-$OSO_3H$ is treated with sodium hydroxide solution to convert the sulfonic acid into the sodium salt and afford monomer **3** (1.62 g, yield 73.50 %). FT-IR (KBr, Figure S3), υ= 3100 $cm^{-1}$ (=C–H), 2905 $cm^{-1}$ (C–H), 1736 $cm^{-1}$ (C=O ester), 1480 $cm^{-1}$ (C=C), 1330 $cm^{-1}$ (O=S=O), 1102 $cm^{-1}$ (C–O), 831 $cm^{-1}$ (C–S). $^1$H-NMR ($D_2O$, 400 MHz, Figure S4): δ= 7.38 (s, 1H, thiophene moiety), 7.23 (d, 1H, thiophene moiety), 7.01 (d, 1H, thiophene moiety), 3.34 (s, 2H, -$CH_2$), 3.55-4.22 (m, 4H, $OCH_2CH_2$ in PEG unit).

### 1.2.5. Synthesis of quinolin-8-yl 2-(thiophen-3-yl)acetate

3-thiopheneacetic acid (1.42 g, 10 mmol), 8-Hydroxyquinoline (1.45 g, 10 mmol), DCC (2.06 g, 10 mmol) and DMAP (0.488 g, 4 mmol) were dissolved in anhydrous DCM (40 mL) and stirred at room temperature for 24 h. The mixture was centrifuged and the supernatant was concentrated in vacuo (20 mbar, 30 °C) redissolved in DCM (10 mL), and washed with 1 mM HCl (pH 3) (3 × 30 mL) saturated $NaHCO_3$ (3 × 30 mL), and $H_2O$ (3 × 30 mL). The organic phase was dried over $MgSO_4$ for 12 h, filtered, and concentrated in vacuo (20 mbar, 30 °C) to give quinolin-8-yl 2-(thiophen-3-yl) acetate as a brown solid (2.32 g, yield 86.24 %). FT-IR (KBr, Figure S5), υ= 3090 $cm^{-1}$ (=C–H), 2955 $cm^{-1}$ (C–H), 1748 $cm^{-1}$ (C=O ester), 1610 $cm^{-1}$ (C=C benzene), 1120 $cm^{-1}$ (C–N), 782 $cm^{-1}$ (C–S). $^1$H-NMR ($D_2O$, 400MHz, Figure S6): δ= 7.66-8.93 (m, 6H, quinoline moiety), 7.51 (s, 1H, thiophene moiety), 7.45 (d, 1H, thiophene moiety), 7.20 (d, 1H, thiophene moiety), 3.50 (s, 2H, -$CH_2$).

**1.2.6. Synthesis of 1-octyl-8-(2-(thiophen-3-yl)acetoxy)quinolin-1-ium bromide (monomer 4)**

1-Bromooctane (0.193 g, 1 mmol) was dissolved in 20 mL of $CH_2Cl_2/CH_3OH$ (v/v = 3/2) with the subsequent addition of quinolin-8-yl 2-(thiophen-3-yl)acetate (0.276 g, 1.3 mmol). The mixture was stirred at room temperature for 24 h. After the reaction was completed, the reaction solution was concentrated to 5 mL. The residue was poured into 200 mL of absolute diethyl ether under stirring and then filtered. The precipitate was washed with absolute diethyl ether and dried to give compound **monomer 3** (0.39 g, yield 83.15%) as a yellow powder. FT-IR (KBr, Figure S7), υ= 3050 $cm^{-1}$ (=C–H), 2960 $cm^{-1}$ (C–$H_{asy}$), 2875 $cm^{-1}$ (C–H sy), 1750 $cm^{-1}$ (C=O ester), 1590 $cm^{-1}$ (C=C benzene), 1360 $cm^{-1}$ ($(CH_2)_n$), 1100 $cm^{-1}$ (C–N), 794 $cm^{-1}$ (C–S). $^1$H-NMR ($D_2O$, 400 MHz, Figure S8): δ= 7.98-8.96 (m, 6H, quinoline moiety), 7.81 (s, 1H, thiophene moiety), 7.35 (d, 1H, thiophene moiety), 7.31 (d, 1H, thiophene moiety), 3.45 (s, 2H, -$CH_2$), 3.21-2.11(m, 14H, alkyl chain), 1.02 $cm^{-1}$ (t, 3H, -$CH_3$).

**1.2.7. Synthesis of CdTe QDs**

The synthesis of CdTe QD was carried out according to the procedure described in the literature with small modifications [1]. Briefly, Te powder (0.1 mmol), $NaBH_4$ (0.2 mmol) and 0.5 mL of water were mixed and heated at 55 °C for 30 min until the black Te disappeared and the pink color of the NaHTe precursor was produced. At the same time $CdCl_2$ (1 mmol), MAA (2 mmol), and 100 mL of distilled water were mixed in a three-neck flask to form a cadmium precursor. The pH of the solution was adjusted to 10.5 using 1.0 N NaOH under vigorous stirring. The mixture was heated to 100 °C under stirring. Meanwhile, the NaHTe precursor solution was quickly injected into the cadmium precursor solution. The molar ratio of $Cd^{2+}$: $Te^{2-}$: MAA was 1:0.1:2. The reaction medium was refluxed at 100 °C for 4 h and then cooled to room temperature. CdTe QDs were precipitated from the solution by added isopropanol at the volume ratio of 1:1 (isopropanol: water) followed by centrifugation at 4000 rpm.

**1.2.8. Synthesis of CdTe QDs coated with cationic, nonionic, anionic polythiophenes and thiophene copolymer via in situ polymerization in aqueous solution (CPTQDs, NPTQDs, APTQDs, and TCPQDs)**

CPTQDs, NPTQDs and APTQDs were synthesized according to our previously reported procedures with some modification [2]. Briefly, each of monomers (1, 2 or 3) (0.03 mmol) was dissolved in 15 mL aqueous solution. The resulted solution was added to 5 mL of CdTe QDs (pH 7) and stirred for 30 min under a nitrogen atmosphere, then $(NH_4)_2S_2O_8$ (0.6 mmol) was added drop-wise into the mixture. The mixture was stirred for 24 h at 25 ± 1 °C. The same procedure was carried out for the synthesis of TCPQDs using monomer 1 (0.03 mmol), monomer 2 (0.04 mmol) and monomer 4 (0.004 mmol). The CPTQDs, NPTQDs, APTQDs and TCPQDs were precipitated by adding 20 mL of methanol. The precipitated materials were recovered by filtration and washed for 3 h by stirring in acetone to remove oligomers and initiator residues. The resulted polymers were air-dried overnight, followed by drying under vacuum (Scheme 1B).

**CPTQDs:** FT-IR (KBr, Figure S9-a), υ= 3320 $cm^{-1}$ (N–H), 2910 $cm^{-1}$ (C–H asy), 2853 $cm^{-1}$ (C–H sy), 1652 $cm^{-1}$ (C=O amide), 1533 $cm^{-1}$ (N-H bond), 1480 $cm^{-1}$ (C=C), 1180 $cm^{-1}$ (C–N), 780 $cm^{-1}$ (C–S). $^1$H-NMR ($D_2O$, 400 MHz, Figure S10): δ= 8.21 (t, 1H, N-H), 7.25 (s, 1H, thiophene moiety), 3.44 (s, 2H, -$CH_2$), 3.11 (m, 2H, -$CH_2$), 3.01 (m, 2H, -$CH_2$), 2.75 (s, 6H, -$CH_3$), 2.22-1.29 (m, 14H, alkyl chain), 0.85 $cm^{-1}$ (t, 3H, -$CH_3$).

**NPTQDs:** FT-IR (KBr, Figure. S9-b), υ= 3410 $cm^{-1}$ (O–H), 2922 $cm^{-1}$ (C–H), 1739 $cm^{-1}$ (C=O ester), 1492 $cm^{-1}$ (C=C), 1150 $cm^{-1}$ (C–O), 810 $cm^{-1}$ (C–S). $^1$H-NMR ($D_2O$, 400 MHz, Figure. S11): δ= 7.22 (s, 1H, thiophene moiety), 3.65 (s, 2H, -$CH_2$), 3.70-4.26 (m, 4H, $OCH_2CH_2$ in PEG unit), 2.08 (t, 1H, -OH).

**APTQDs:** FT-IR (KBr, Figure S9-c), 2960 $cm^{-1}$ (C–H), 1750 $cm^{-1}$ (C=O ester), 1580 $cm^{-1}$ (C=C,), 1370 $cm^{-1}$ (O=S=O), 1170 $cm^{-1}$ (C–O), 885 $cm^{-1}$ (C–S). $^1$H-NMR ($D_2O$, 400 MHz, Figure. S12): δ= 7.29 (s, 1H, thiophene moiety), 3.32 (s, 2H, -$CH_2$), 3.41-4.31 (m, 4H, $OCH_2CH_2$ in PEG unit).

**TCPQDs:** FT-IR (KBr, Figure S9-d), υ= 3500 $cm^{-1}$ (O–H), 3350 $cm^{-1}$ (N–H), 3090 $cm^{-1}$ (=C–H), 2980 $cm^{-1}$ (C–H asy), 2887 $cm^{-1}$ (C–H sy), 1745 $cm^{-1}$ (C=O ester), 1650 $cm^{-1}$ (C=O amide), 1500 $cm^{-1}$ (N-H bond), 1200 $cm^{-1}$ (C–O), 1050 (C–N), 767 $cm^{-1}$ (C–S). $^1$H-NMR ($D_2O$, 400 MHz, Figure S13): δ= 7.33-9.12 (m, 6H, quinoline moiety), 8.33 (t, 1H, N-H), 7.11 (s, 1H, thiophene moiety), 6.95 (s, 1H, thiophene moiety), 6.77 (s, 1H, thiophene moiety), 4.31 (s, 2H, -$CH_2$), 3.55-4.66 (m, nH, $OCH_2CH_2$ in PEG unit), 2.56 (s, 6H, -$CH_3$), 2.11 (t, 1H, -OH), 1.36-0.91(m, nH, alkyl chain), 0.91 $cm^{-1}$ (t, 3H, -$CH_3$).

**1.3. The determination of quantum yield (QY)**

The quantum yields of CPTQDs, NPTQDs, APTQDs and TCPQDs were obtained by comparing the integrated FL intensities and the absorbance values of the QDs with the reference, rhodamine B (Φ = 0.31), and the as-prepared QDs were dissolved in water (n = 1.33). A UV−vis absorption spectrometer was used to determine the absorbance values of the samples at 340, 350, 360 and 380 nm excitation wavelengths, respectively. The spectrophotometer set with an excitation slit width of 3 nm and an emission slit width of 3 nm was used to excite the samples to record their FL spectra. The QY was calculated using the equation (1) below [3].

$$\Phi_x = \Phi_r \times \frac{I_x}{I_r} \times \frac{A_x}{A_r} \times \frac{n_x^2}{n_r^2} \qquad (1)$$

where $\Phi_x$ is the QY, I is the integrated fluorescence intensity, A is the absorbance, and n is the refractive index of the solvent; r denotes the standard and x denotes the sample.

**1.4. Biocompatibility**

To assess the biocompatibility of the CPTQDs, NPTQDs, APTQDs and TCPQDs in comparison with equivalent content of bare CdTe QDs, an MTT cell assay was performed on the HeLa cells. Briefly, HeLa cells were plated at a density of $1\times10^4$ cells per well in a 96-well plate, and then incubated for 24 h at 37 °C under 5% $CO_2$ to allow the cells to attach to the wells. The CPTQDs, NPTQDs, APTQDs and TCPQDs were sterilized by autoclaving, and two different concentration (400 and 600 µg/mL) from QDs coated amphiphilic polymers were added to the culture wells to replace the original culture medium and were incubated for another 24 h in 5% $CO_2$ at 37 °C. For comparison the equivalent content of bare CdTe QDs (Table S3) were calculated based on TGA results (Figure 1d) and were added to the culture wells to replace the original culture medium and were incubated for another 24 h in 5% $CO_2$ at 37 °C. Next, 10 µL of MTT solution (5mg/mL) was added to each well (containing different amounts of the CPTQDs, NPTQDs, APTQDs and TCPQDs and their equivalent of CdTe QDs, followed by incubation for 4 h inside a $CO_2$ incubator at 37 °C. After incubation, the medium was removed, and the formed formazan crystals were dissolved in 100 µL of

DMSO/ethanol mixture (1:1). A Tecan Infinite M200 monochromator-based multifunction microplate reader was used to measure the OD 570 (Abs value) of each well with background subtraction at 540 nm. At least three independent experiments were performed in each case. The following equation (2) was applied to calculate the viability of cell growth [4] :

$$\text{Cell viability (\%)} = \frac{\text{mean Abs value of treatment group}}{\text{mean Abs value of control}} \times 100 \qquad (2)$$

**1.5. Fluorescence sensing of TNP explosive**

Stock solutions (0.01 mM) of CPTQDs, NPTQDs, APTQDs, and TCPQDs were prepared in PBS buffer (pH 7.0). Different concentrations of TNP were mixed with 1.5 mL of the respective sensor solutions such that the total volume amounted to 3 mL, and the samples were incubated for 60 s at room temperature before detection. The fluorescence spectra of the samples were measured with excitation at 340, 350, 360, and 380 nm, for the CPTQDs, NPTQDs, APTQDs, and TCPQDs, respectively. All the measurements were carried out in triplicate.

**1.6. Determination of selectivity to TNP**

This method entailed selecting several nitroaromatic explosives (Scheme S1) and metal ions as coexisting substances to investigate the selectivity of TNP. The concentration of each explosive was 50 μM, whereas the concentration of metal ions was selected as 1 mM. The selected detection conditions were the same as those mentioned above.

**1.7. TRPL studies**

Excited state lifetime decay profiles of CPTQDs, NPTQDs, APTQDs and TCPQDs were obtained in both solvents before and after TNP addition via 375 nm pulse excitation and emission at 425, 430, 460, and 510 nm in aqueous medium, respectivly. The decay profiles were bi-exponentially fitted and for uniformity in results average lifetime were considered.

**1.8. Determination of TNP in enviromental water samples**

River and tap water samples were utilized to investigate the practical applicability of the developed method for the detection of TNP. Samples of river water were collected from the Changwon River (South Korea). Both types of water samples were filtered twice to remove any solid suspensions and then centrifuged at 4000 rpm for 15 min. The spiked river and tap water samples were diluted 50-fold with different concentrations of TNP (0.04, 0.80, and 1.60 μM) and added to 0.01 mM solutions of the CPTQD, NPTQD APTQD, and TCPQD sensors, respectively. The samples were incubated for 60 s at a pH of 7.0 before detection and

each experiment was repeated five times under the same conditions to determine the relative standard deviations (RSDs).

**1.9. Preparation of the paper- based sensor**

Whattman filter paper (70 mm diameter) was immerged in the TCPQDs ($10^{-4}$ M) solution for 60 min. The filter paper was then removed from the solution and dried at room temperature. TCPQDs coated filter papers were then cut into desired number of pieces (1 cm × 1 cm) and a 10 μL of TNP solution with various concentrations was dropped into the obtained filter paper strips, and the solvent on the filter paper strips was naturally evaporated at room temperature. Paper strips were then visualized under 365 nm UV light. Furthermore, fluorescence color image was taken with a smartphone, and then RGB intensities of the image can be directly output by using a custom developed PAD Analysis APP, which can be readily downloaded to the smartphone online.

**1.10. Preparation of fluorescence film-based sensor**

In a 50 mL beaker containing TCPQDs (15 mg) and purified chitosan (300 mg), 30 mL Milli-Q water and 300 μL of acetic acid were added followed by continuous stirring for about 15 min to ensure complete dissolution of chitosan. This leads to the formation of highly viscous liquid that was spread on pre-cleaned glass plate/petri dish and dried at room temperature. A homogeneous transparent film was obtained that could be easily lifted using forceps for sensing purposes. For contact mode sensing, 5 mg of TNP was rubbed with the left hand thumb and brushed properly to remove all visible TNP particles. Left thumb was then pressed onto the film for 10 sec, kept aside and the impression observed under UV light. Right hand thumb was used as control without using TNP.

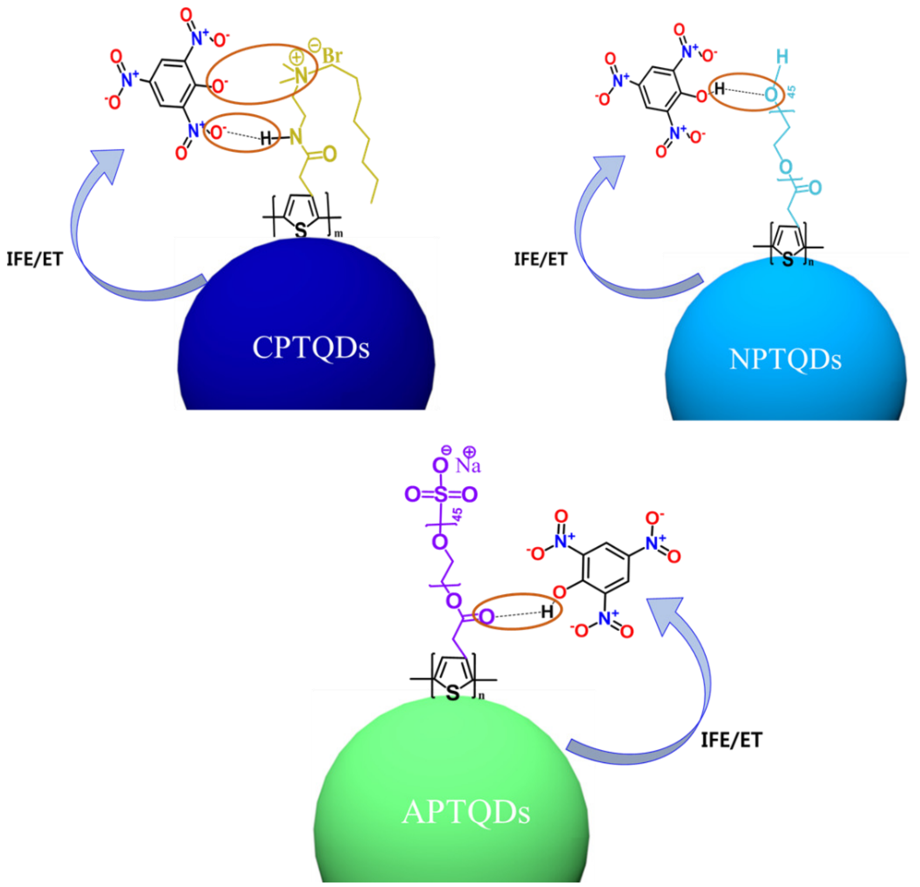


**Scheme S2**. Schematic representation the detection of TNP using the CPTQDs, NPTQDs, and APTQDs sensor through IFE and molecular interactions mechanism.

**Table S1**. Characterization of different amphiphilic conjugated polythiophenes coated QDs

| Samples | Size, nm | ζ potential, mV | $\lambda_{max,abs}$[a] (nm) | $\lambda_{max,em}$[a] (nm) | QY[b] (%) |
|---|---|---|---|---|---|
| CdTe QDs | 4.8 | -35 | 560 | 590 | 5.6 |
| CPTQDs | 39.0 | 18.5 | 340 | 425 | 75 |
| NPTQDs | 43.3 | 2.1 | 350 | 430 | 61 |
| APTQDs | 32.8 | -22.1 | 360 | 460 | 72 |
| TCPQDs | 50.5 | 39.8 | 380 | 510 | 78 |

[a]Measured in aqueous solution with a concentration of 100 μM in water.

[b]Measured in aqueous solution with rhodamine B as standard.

**Table S2**. Percentage elemental composition of the QD nanohybrids from EDX.

| | **CPTQDs** | | **NPTQDs** | | **APTQDs** | | **TCPQDs** | |
|---|---|---|---|---|---|---|---|---|
| Element | Weight % | Atomic % | Weight % | Atomic % | Weight % | Atomic % | Weight % | Atomic % |
| **C** | 36.63 | 54.52 | 53.7 | 58.73 | 31.04 | 42.19 | 50.24 | 56.76 |
| **O** | 30.13 | 27.52 | 34.14 | 31.69 | 21.31 | 30.37 | 20.48 | 20.5 |
| **N** | 10.41 | 6.57 | ----- | ----- | ----- | ----- | 9.12 | 9.14 |
| **S** | 8.43 | 8.71 | 8.26 | 8.28 | 16.52 | 11.35 | 11.06 | 9.27 |
| **Cd** | 2.2 | 0.2 | 1.7 | 0.8 | 2.24 | 0.24 | 1.7 | 0.8 |
| **Te** | 5.2 | 0.6 | 2.2 | 0.5 | 12.24 | 0.63 | 2.2 | 0.5 |
| **Na** | ----- | ----- | ----- | ----- | 16.65 | 15.22 | ----- | ----- |
| **Br** | 7 | 1.88 | ----- | ----- | ----- | ----- | 5.2 | 3.03 |

**Table S3**. Concentration of QDs coated with amphiphilic polythiophenes and their equivalent bare QDs applied for cytotoxicity test.

| **Samples** | **Concentration (µg/mL)** | **Equivalent of bare QDs calculated from TGA results (µg/mL)** |
|---|---|---|
| **CPTQDs** | **400** | **324** |
| | **600** | **486** |
| **NPTQDs** | **400** | **216** |
| | **600** | **324** |
| **APTQDs** | **400** | **196** |
| | **600** | **294** |
| **TCPQDs** | **400** | **172** |
| | **600** | **258** |

**Table S4**. Comparison of the detection limit of the prepared amphiphilic conjugated polythiophenes coated QDs sensors with other reported TNP sensors.

| **Method** | **Detection Limit (nM)** | **References** |
|---|---|---|
| Tetraphenylethelene Nanosphere | 5.0 | [5] |
| p-phenylenevinylene derivative | 11.0 | [6] |
| Polyfluorene derivative | 57.8 | [7] |
| Polydiacetylene microtube | $4.8\times10^{2}$ | [8] |
| Cationic bispyrene fluorophore, Py-dilM-Py | $1\times10^{3}$ | [9] |
| Self-assembled pentacenequinone derivative | 350 | [10] |
| Eu(III)-based metal organic frameworks | 100 | [11] |
| DNSA-SQ | 70 | [12] |
| $MoS_2$ QDs | 95 | [13] |
| N-GQDs | $9.2\times10^{2}$ | [14] |
| APBA functionalized $CuInS_2$ QDs | 28 | [15] |
| FL-MIPs | 43 | [16] |
| Nph-An organic sensor | $4.7\times10^{2}$ | [17] |
| Four sensors based on amphiphilic conjugated polythiophenes coated CdTe QDs | 2.56, 7.23, 4.12 and 0.56 | This work |

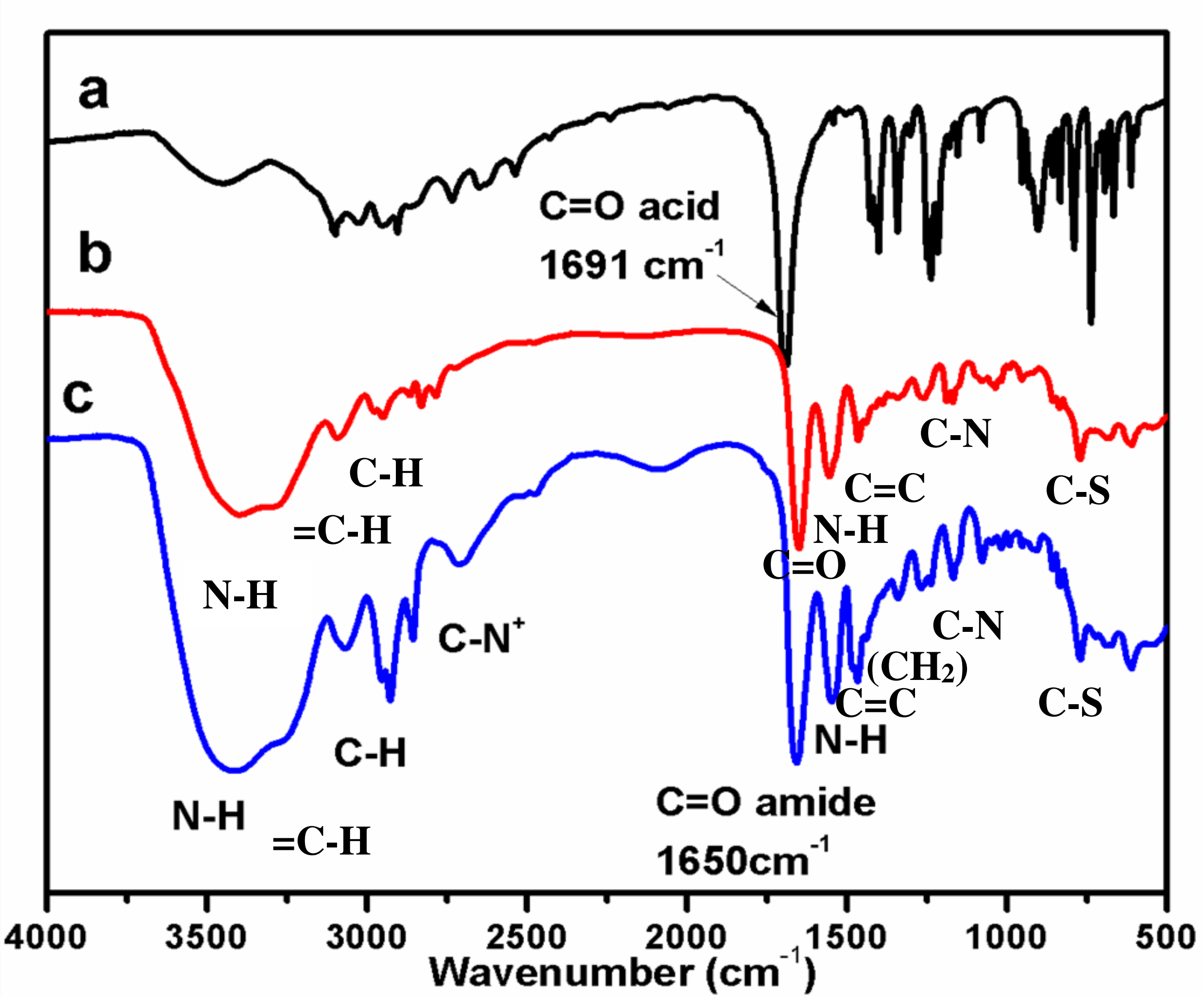


**Figure S1.** FT-IR spectra of cationic monomer (a) 3-thiopheneacetic acid, (b) N-(2-(dimethylamino)ethyl)-2-(thiophen-3-yl)acetamide,(c)N,N-dimethyl-N-(2-(2-(thiophen-3-yl)acetamido)ethyl) octan-1- aminium bromide (**monomer 1**).

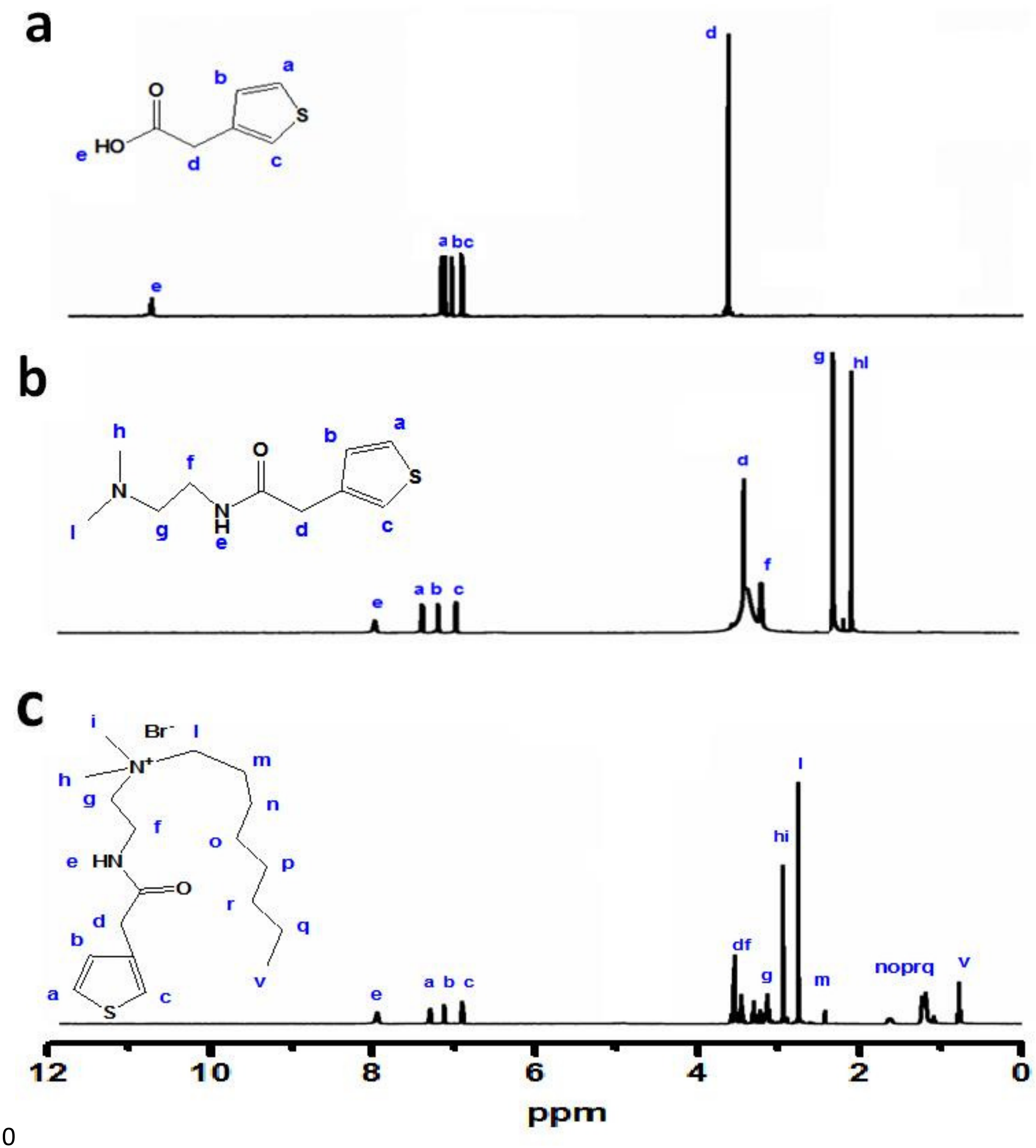


0

**Figure S2.** $^{1}$H-NMR spectra of cationic monomer (a) 3-thiopheneacetic acid, (b) N-(2-(dimethylamino)ethyl)-2-(thiophen-3-yl)acetamide, (c)N,N-dimethyl-N-(2-(2-(thiophen-3-yl)acetamido)ethyl) octan-1- aminium bromide (**monomer 1**).

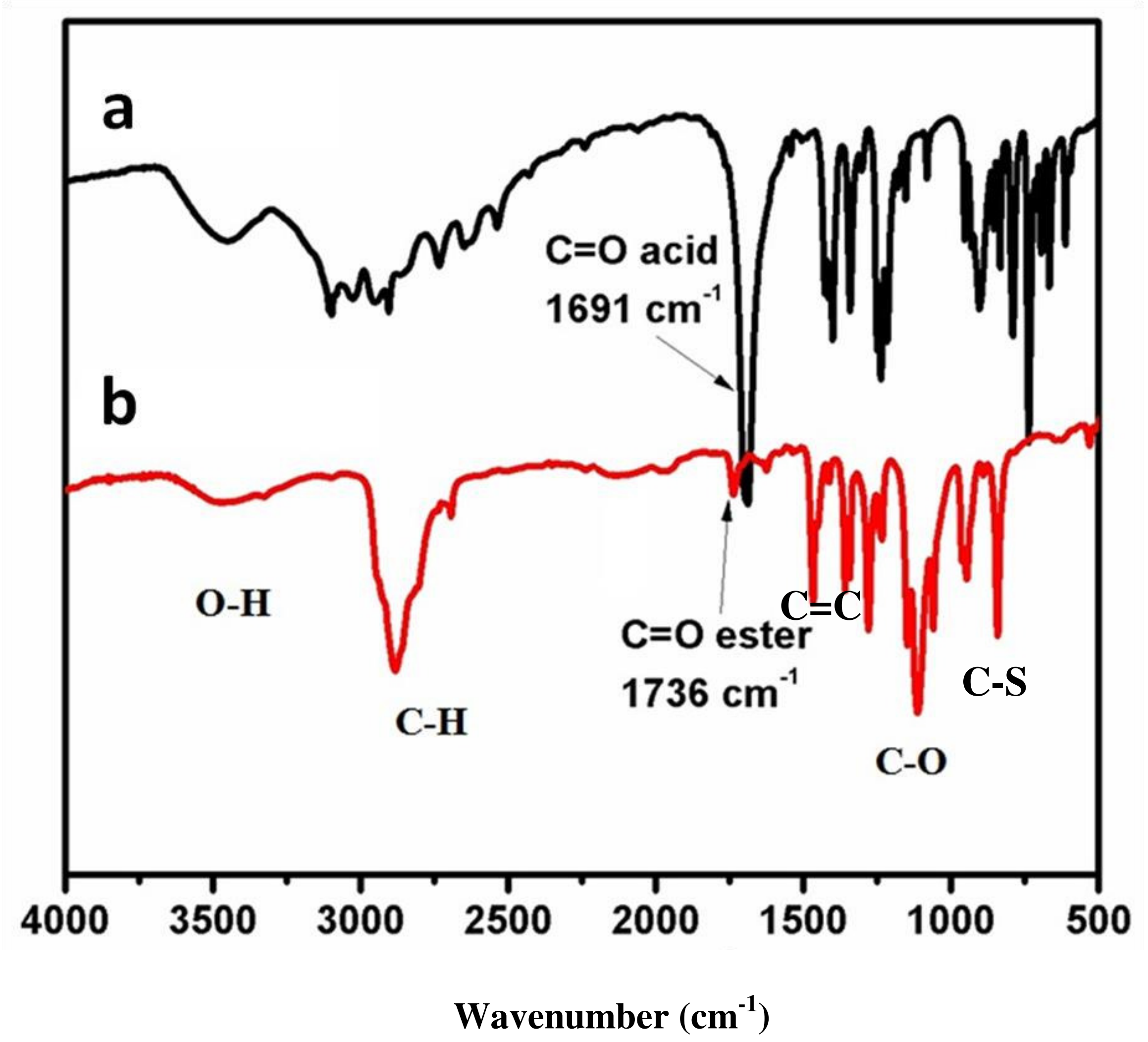


**Figure S3.** FT-IR spectra of nonionic monomer (a) 3-thiopheneacetic acid, (b) 3-thiopheneacetic acid-PEG2000 (**monomer 2**).

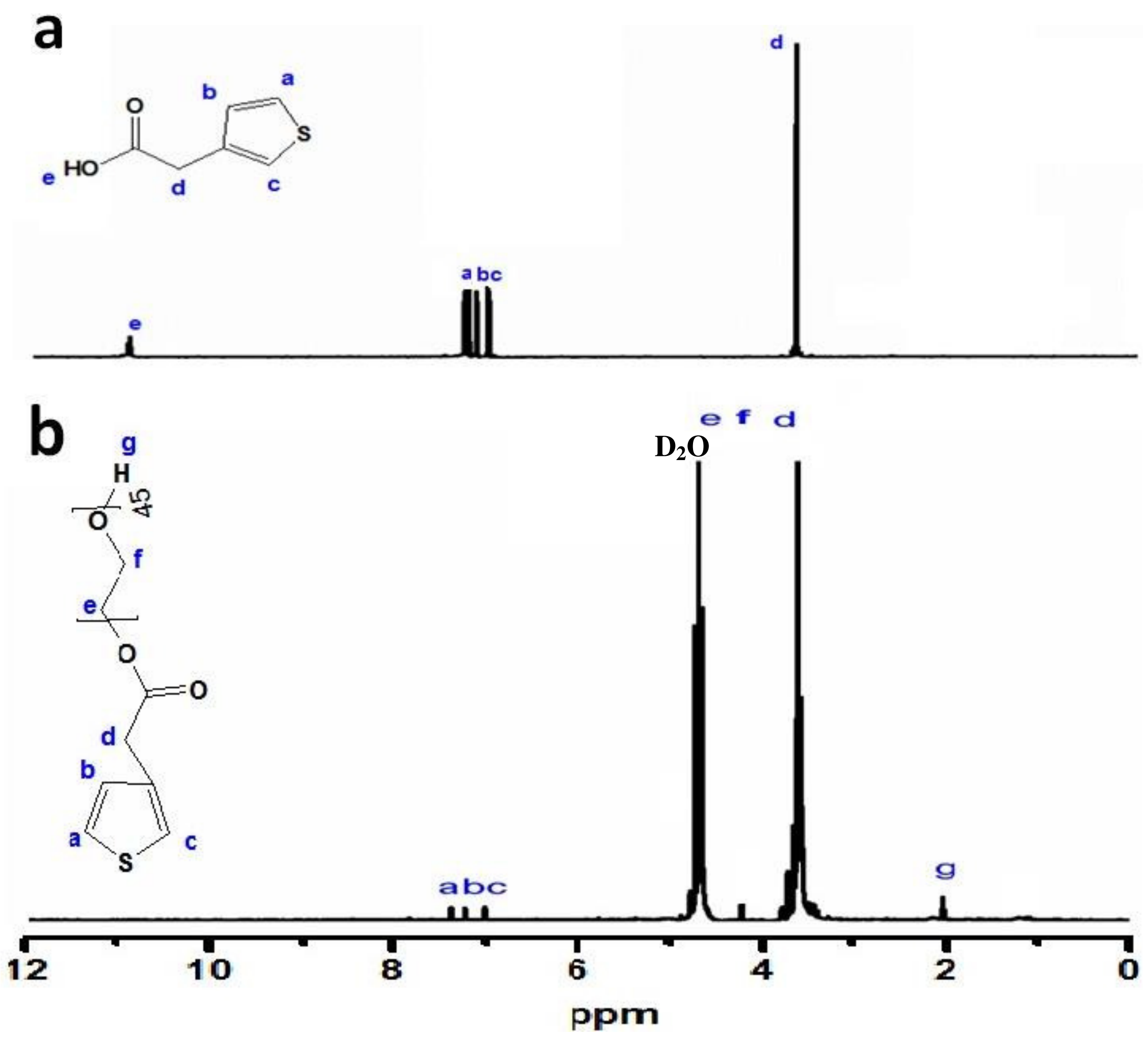


**Figure S4.** $^{1}$H-NMR spectra of nonionic monomer (a) 3-thiopheneacetic acid, (b) 3-thiopheneacetic acid-PEG2000 **(monomer 2)**.

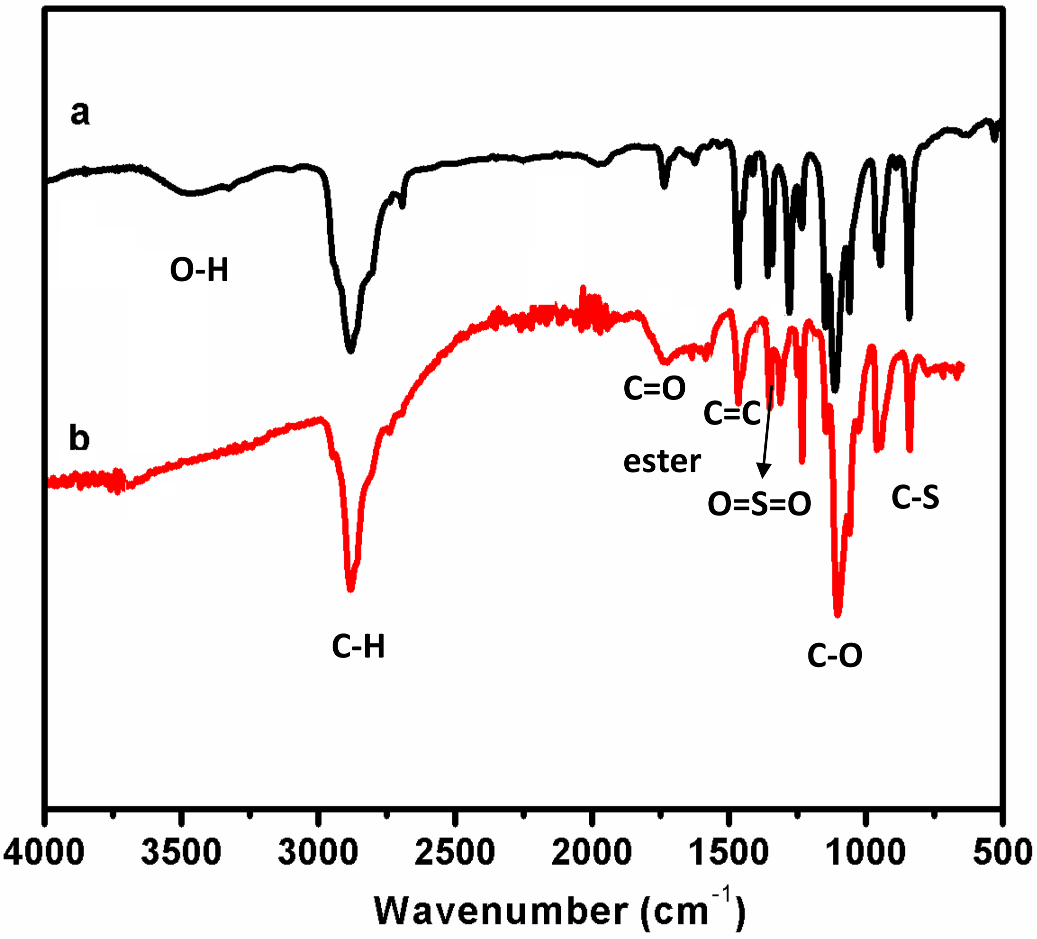


**Figure S5.** FT-IR spectra of anionic monomer (a) 3-thiopheneacetic acid-PEG2000, (b) 3-thiopheneacetic acid-PEG-$OSO_3Na$ **(monomer 3)**.

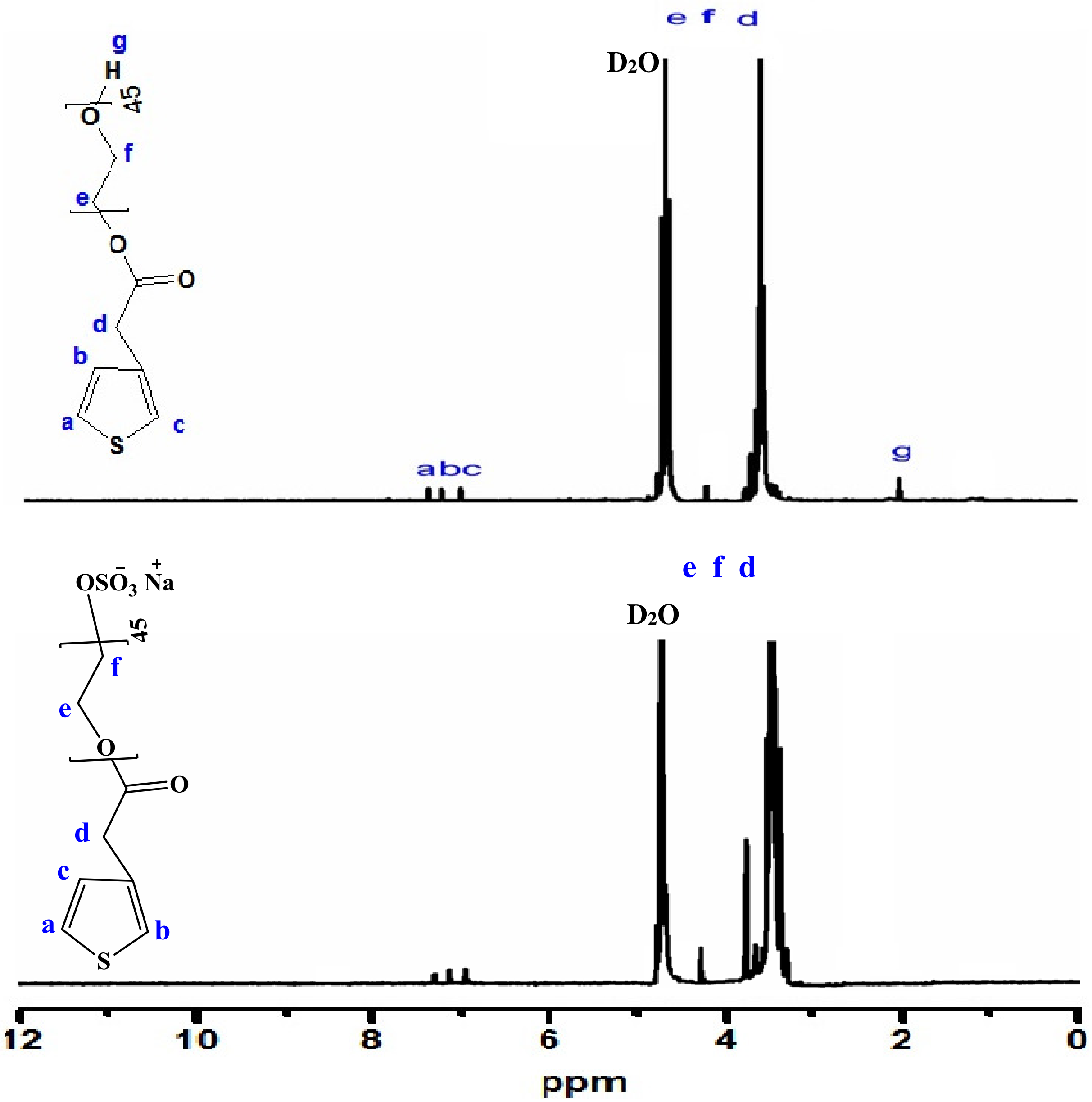


**Figure S6.** $^{1}$H-NMR spectra of anionic monomer (a) 3-thiopheneacetic acid-PEG2000, (b) 3-thiopheneacetic acid-PEG-$OSO_3Na$ (**monomer 3**).

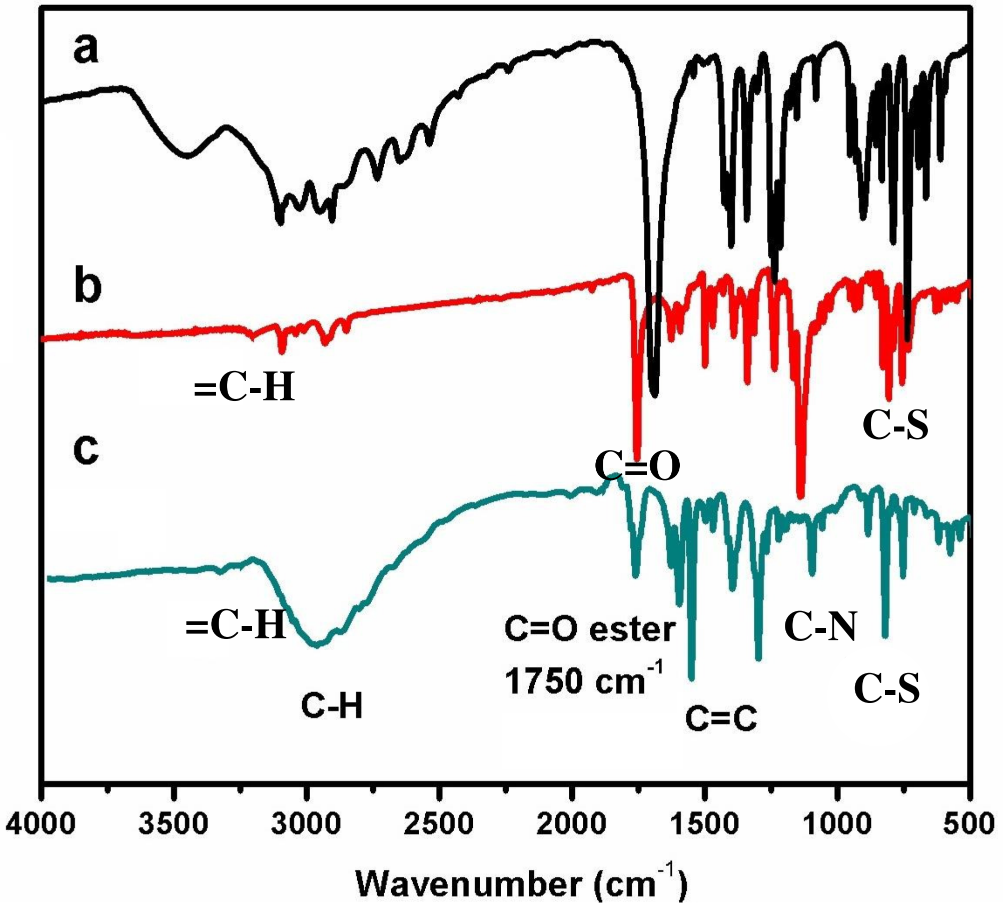


**Figure S7.** FT-IR spectra of conjugated cationic monomer (a) 3-thiopheneacetic acid, (b) quinolin-8-yl 2-(thiophen-3-yl)acetate, (c) 1-octyl-8-(2-(thiophen-3-yl)acetoxy)quinolin-1-ium bromide (**monomer 4**).

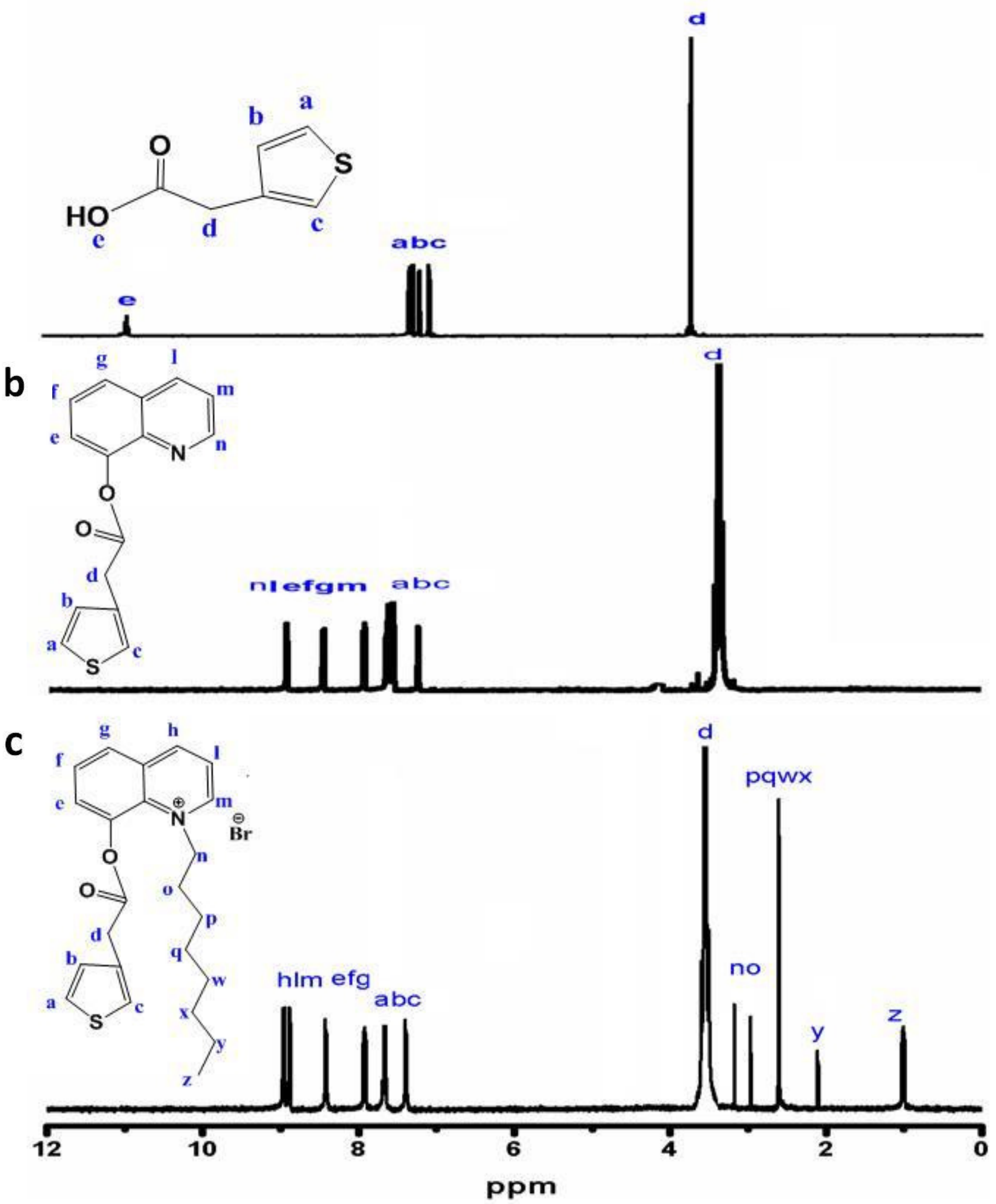


**Figure S8.** $^{1}$H-NMR spectra of conjugated cationic monomer (a) 3-thiopheneacetic acid, (b) quinolin-8-yl 2-(thiophen-3-yl)acetate, (c) 1-octyl-8-(2-(thiophen-3-yl)acetoxy)quinolin-1-ium bromide (**monomer 4**).

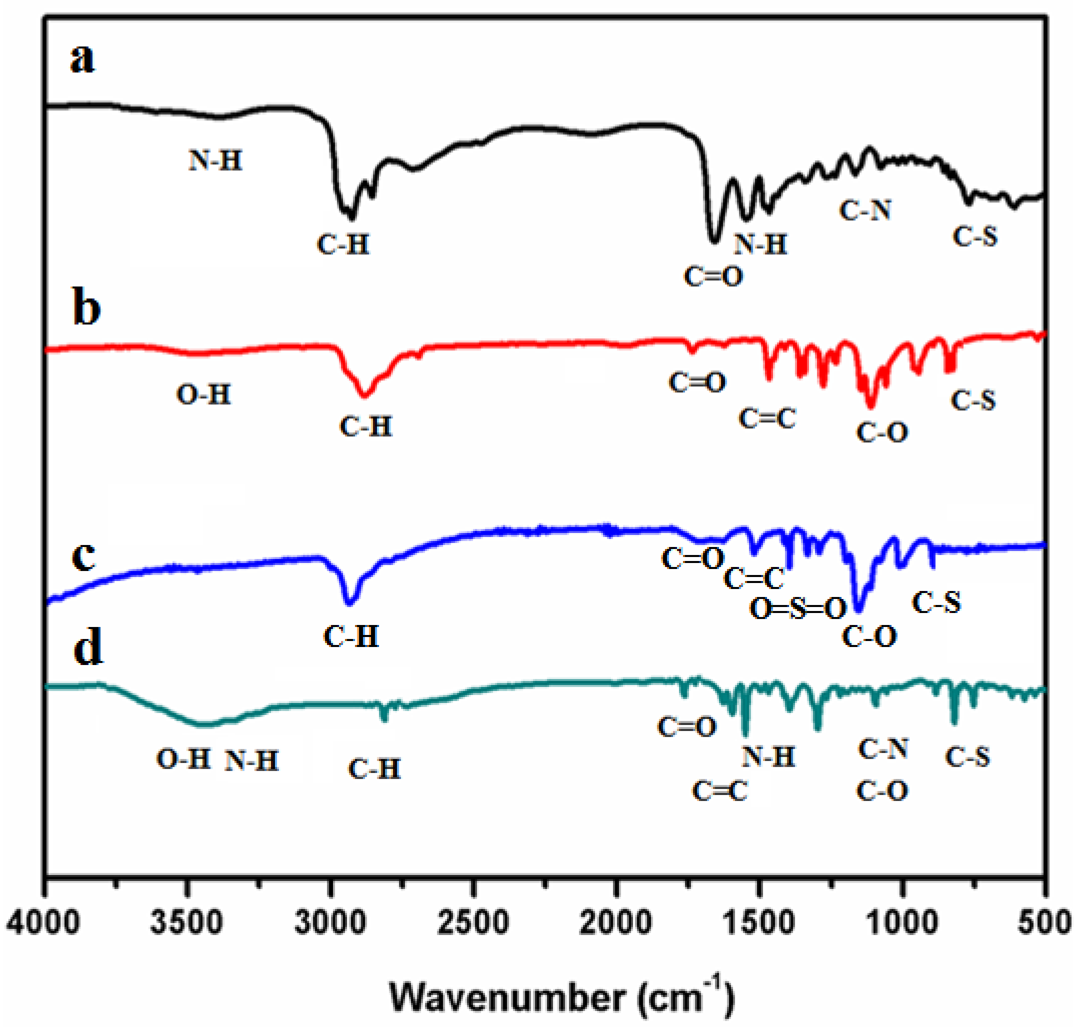


**Figure S9.** FT-IR spectra of (a) **CPTQDs**, (b) **NPTQDs**, (c) **APTQDs** and (a) **TCPQDs** nanohybrids.

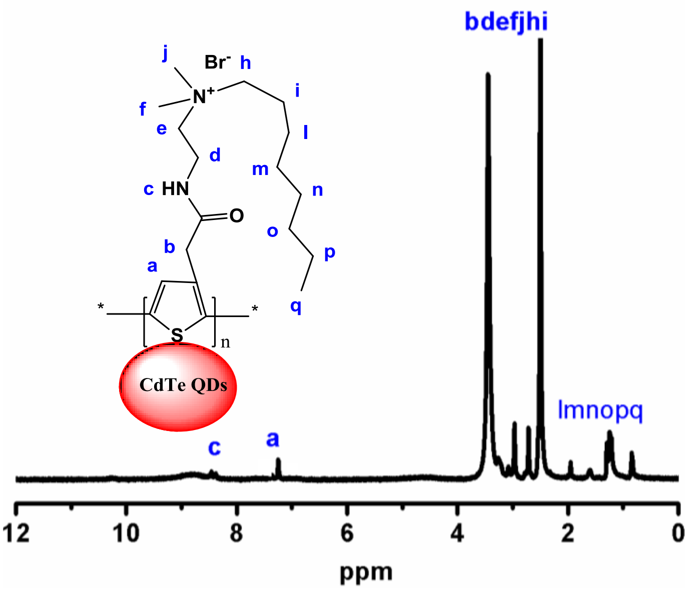


**Figure S10.** $^{1}$H-NMR spectra of CdTe QDs coated with cationic conjugated polythiophene (CPTQDs)

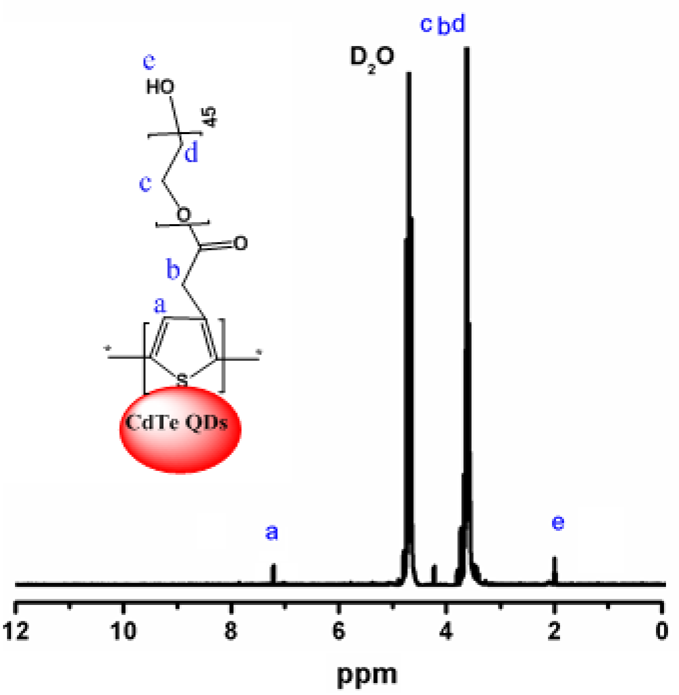


**Figure S11.** $^{1}$H-NMR spectra of CdTe QDs coated with nonionic conjugated polythiophene (NPTQDs).

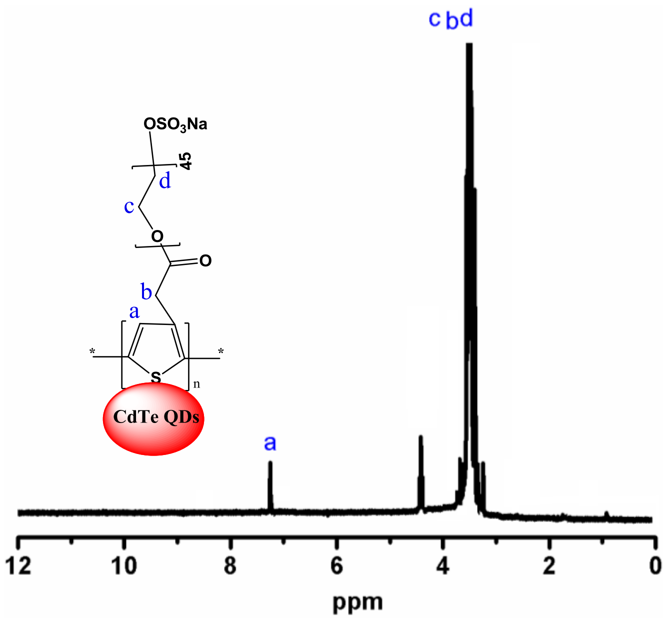


**Figure S12.** $^{1}$H-NMR spectra of CdTe QDs coated with anionic conjugated polythiophene (APTQDs).

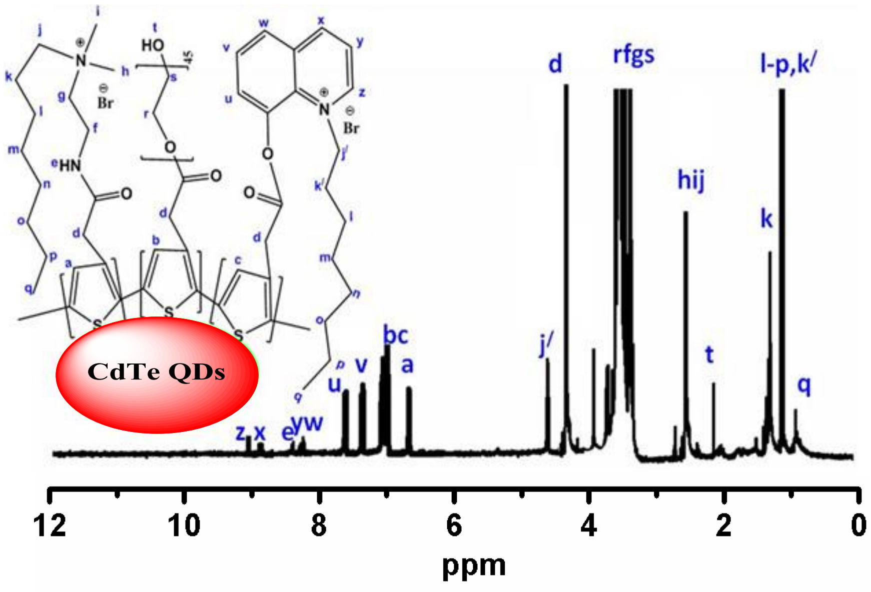


**Figure S13.** $^{1}$H-NMR spectra of CdTe QDs coated with thiophene copolymer (TCPQDs).

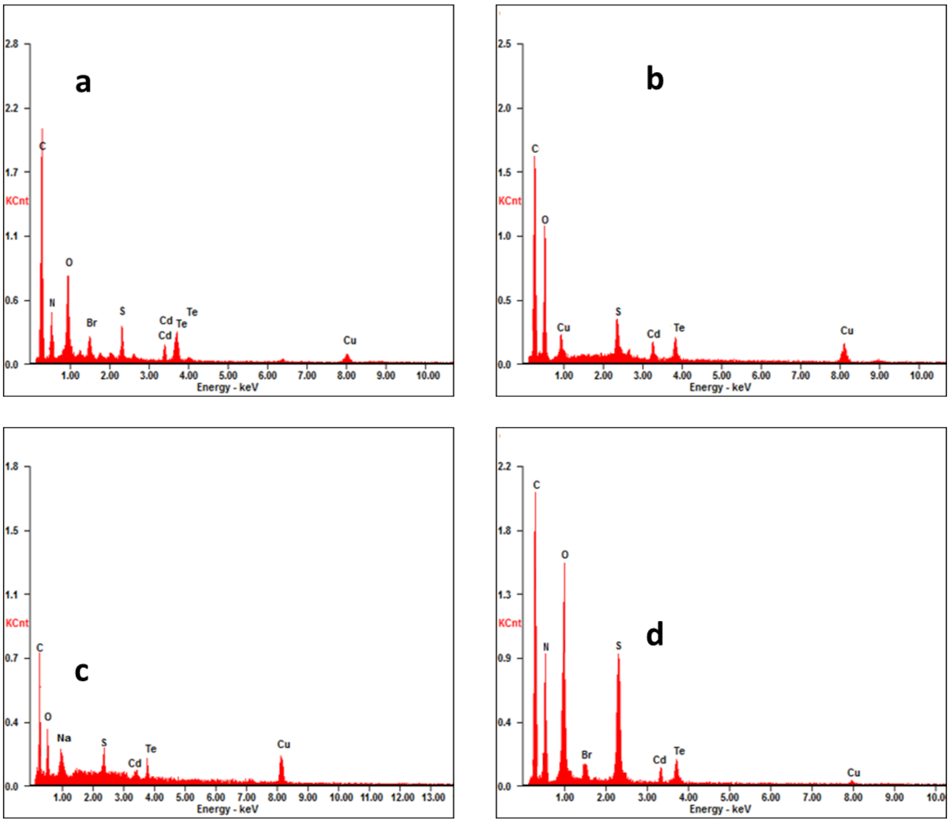


**Figure S14**. EDX spectra of the CPTQDs, NPTQDs, APTQDs and TCPQDs nanohybrids

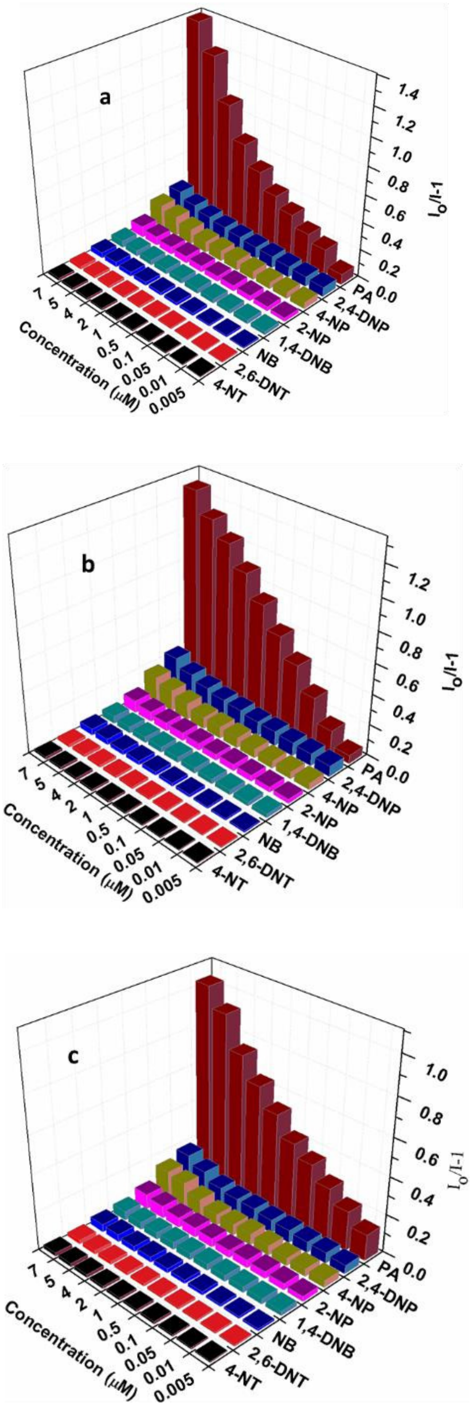


**Figure S15**. The Stern-Volmer plots of CPTQDs (a), NPTQDs (b) and APTQDs (c) with nitroaromatic compounds.

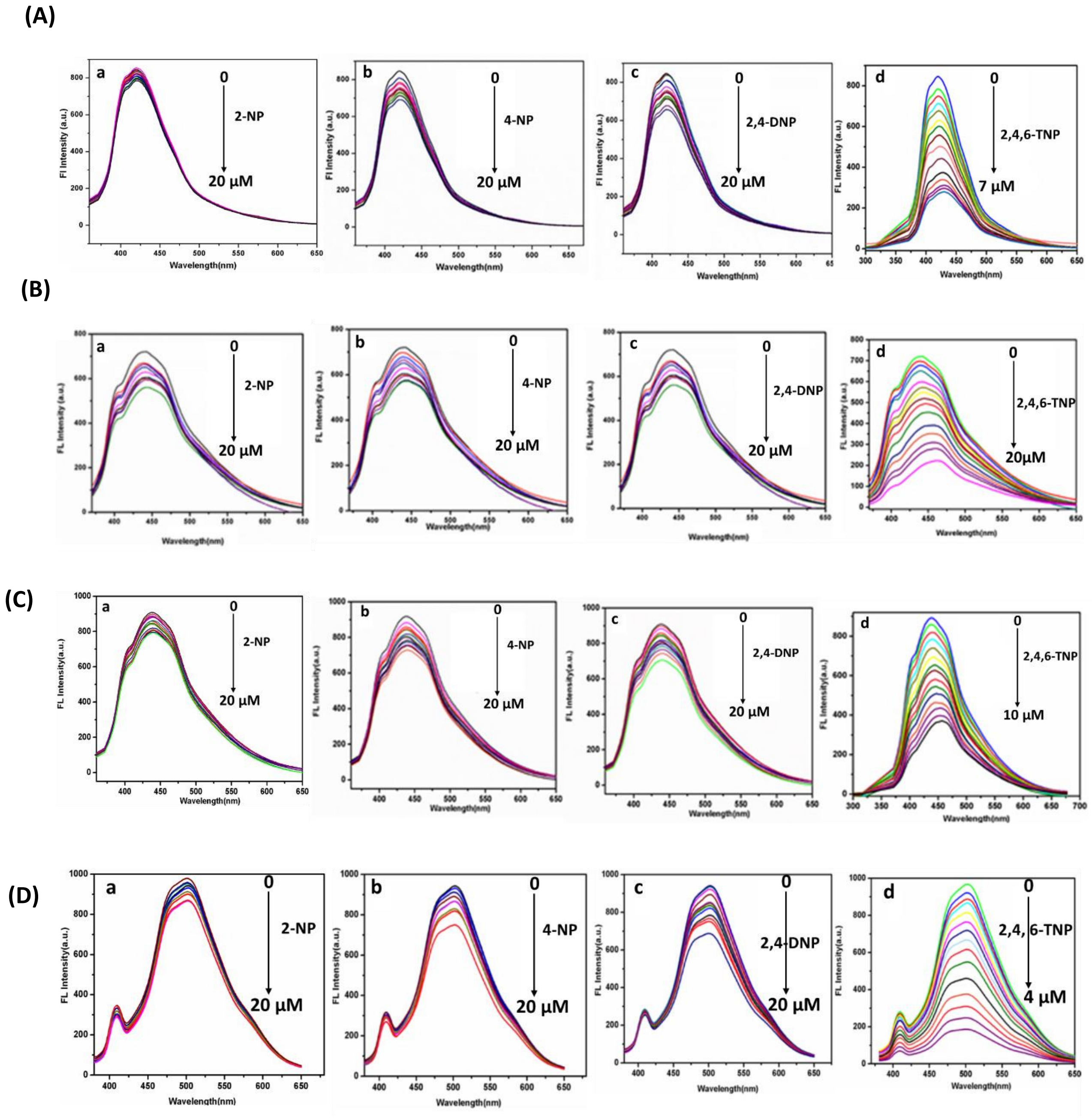


**Figure S16**. Quenching efficiency of (A) CPTQDs, (B) NPTQDs, (C) APTQDs and (D) TCPQDs toward 2-nitrophenol (2-NP, a), 4-nitrophenol (4-NP, b), 2,4-dinitrophenol (2,4-DNP, c), 2,4,6-trinitrophenol (2,4,6-TNP, d). Experimental condition (PBS, pH 7.0), incubation time, 60 sec).

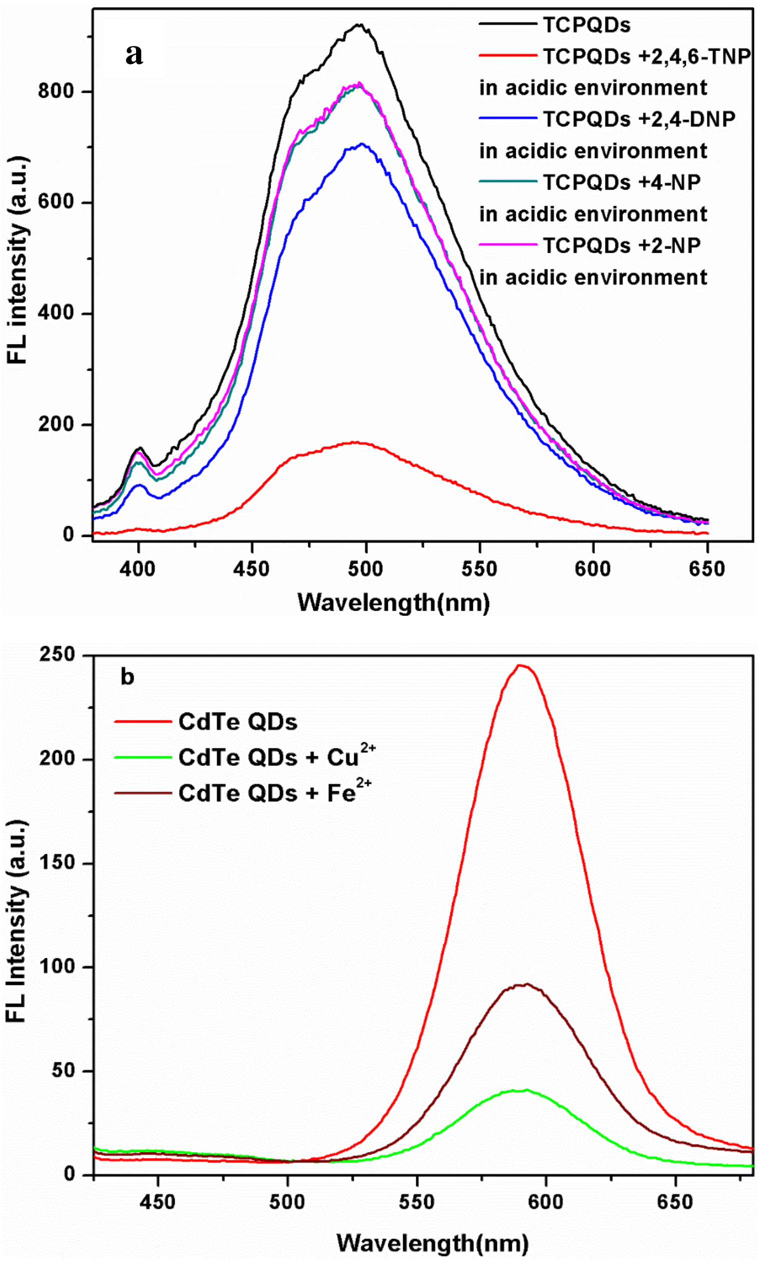


**Figure S17.** Fluorescence spectra of TCPQDs with the addition of 2-nitrophenol (2-NP), 4-nitrophenol (4-NP), 2,4-dinitrophenol (2,4-DNP), 2,4,6-trinitrophenol (2,4,6-TNP) in the same acidic environments (a), and quenching effect of some metal ions (such as $Cu^{2+}$ and $Fe^{2+}$) on the FL intensity of bare CdTe QDs (b).

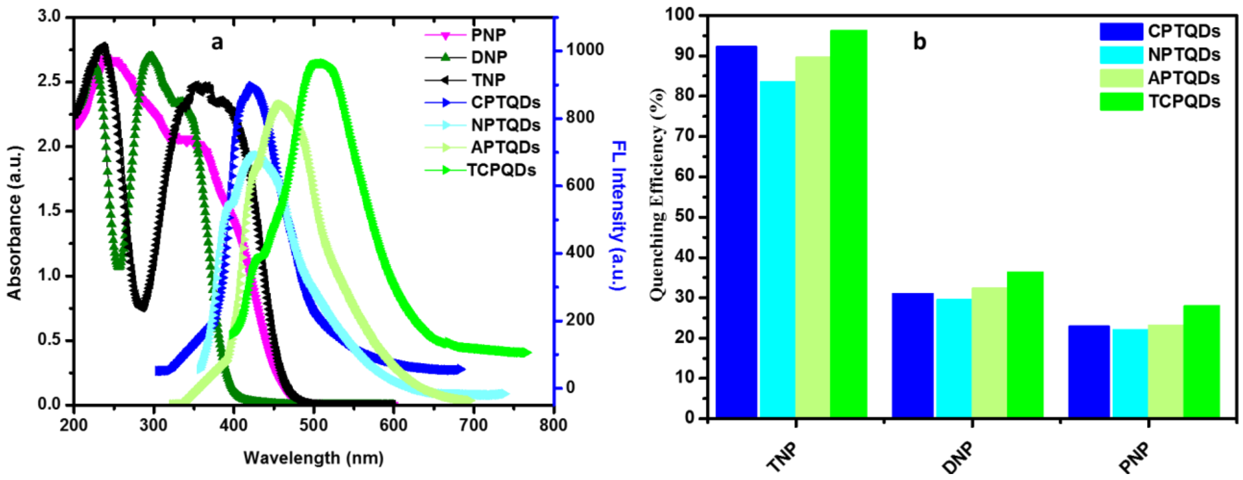


**Figure S18**. (a)The UV-vis absorption spectra of TNP, DNP, PNP and the fluorescence emission spectrum of the CPTQDs, NPTQDs, APTQDs and TCPQDs, (b) Comparison of quenching efficiency for PNP, DNP and TNP in 10 mM PBS (pH 7.0).

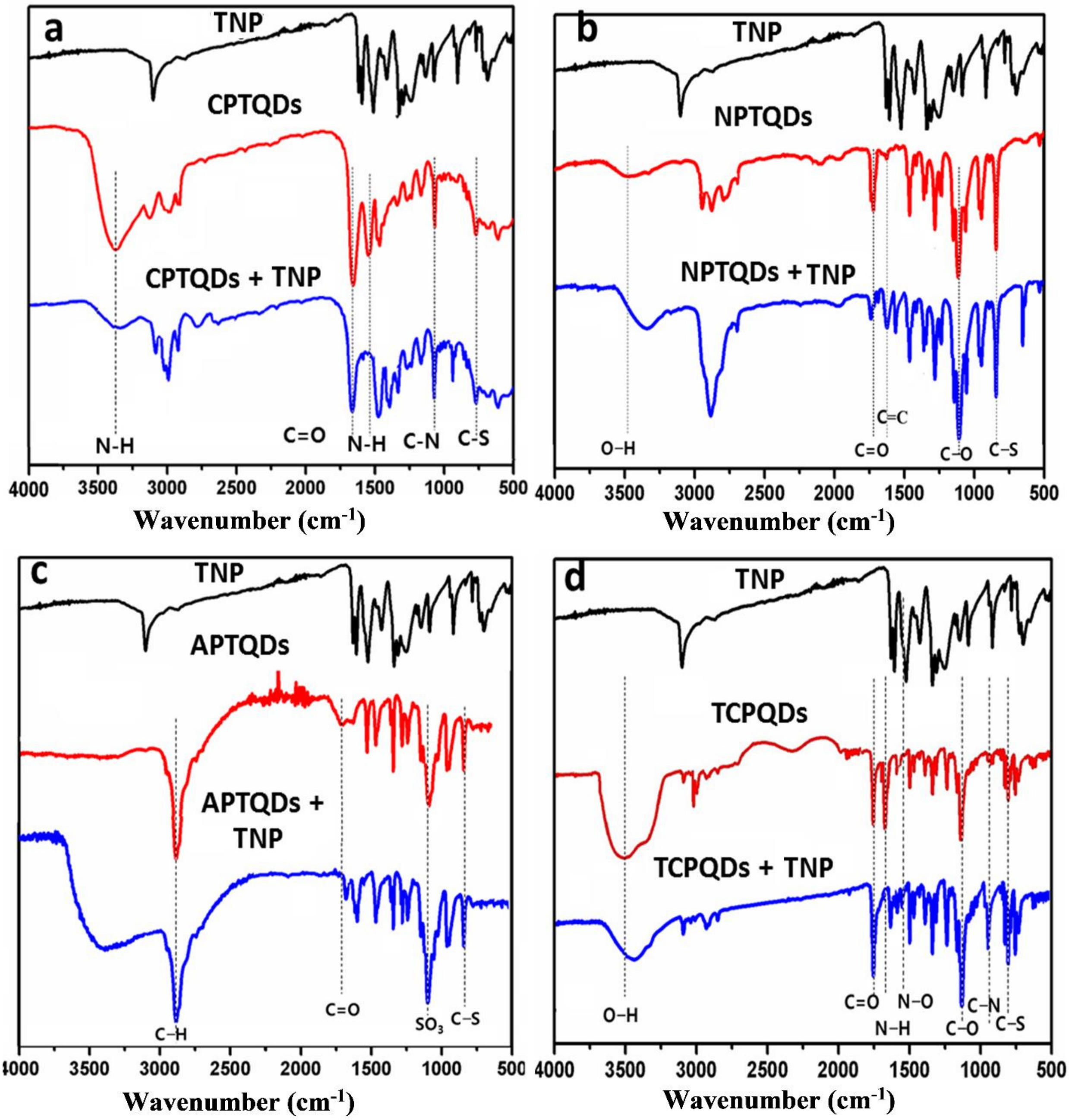


**Figure S19**. FT-IR spectra of (a) TNP, CPTQDs, CPTQDs +TNP, (b) TNP, NPTQDs, NPTQDs + TNP, (c) TNP, APTQDs, APTQDs + TNP and (d) TNP, TCPQDs, TCPQDs + TNP

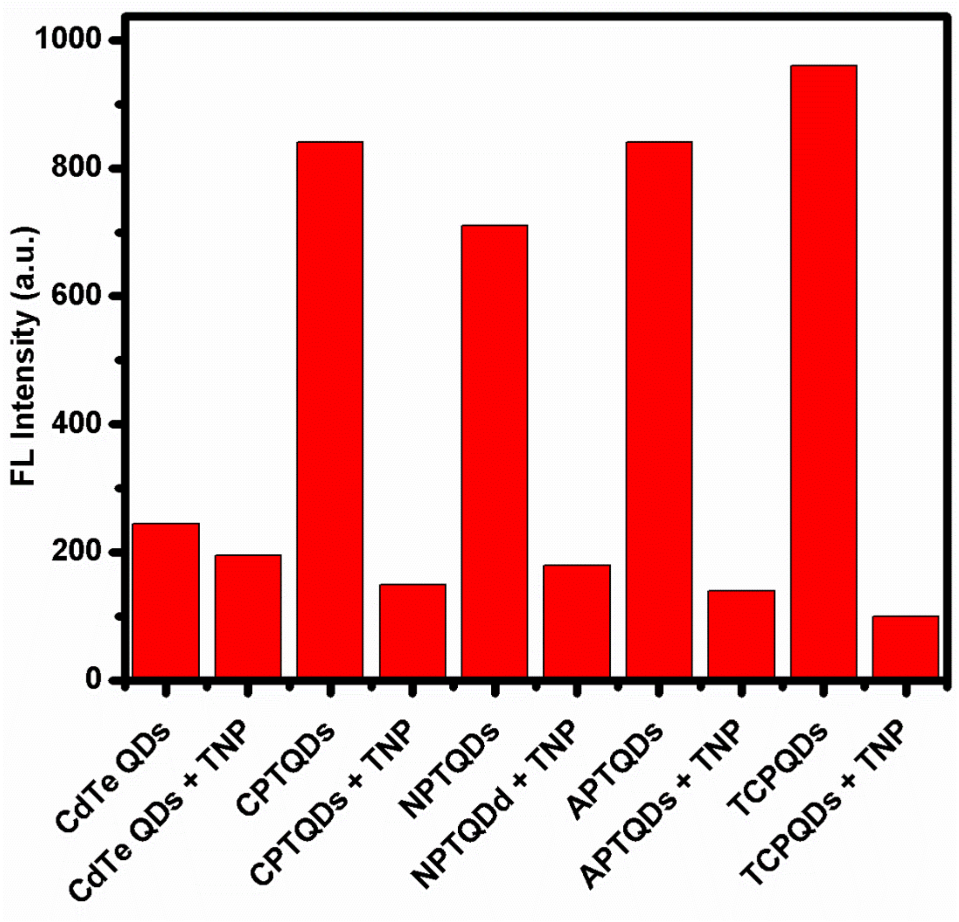


**Figure S20**. Effect of TNP (20 μM) on the fluorescence emission intensity of bare CdTe QDs, CPTQDs, NPTQDs, APTQDs, and TCPQDs

**Reproducibility and stability of the sensors**

The reproducibility of CPTQDs, NPTQDs, APTQDs and TCPQDs was investigated by the measurement of the response to 5 µM from TNP. The relative standard deviations (RSDs) of the ten successive measurements were 1.98, 2.75, 2.04, and 1.62%, respectively, Figures. (S21a-d). In addition, the stability of the sensors was investigated after being stored at 4 °C for more than 120 days. The CPTQDs, NPTQDs, APTQDs and TCPQDs responses were stable and maintained 90, 92, 88% and 95% activity with a low deviation of 5, 4, 5 and 4%, respectively, indicating excellent stability of the sensors for the determination of TNP.

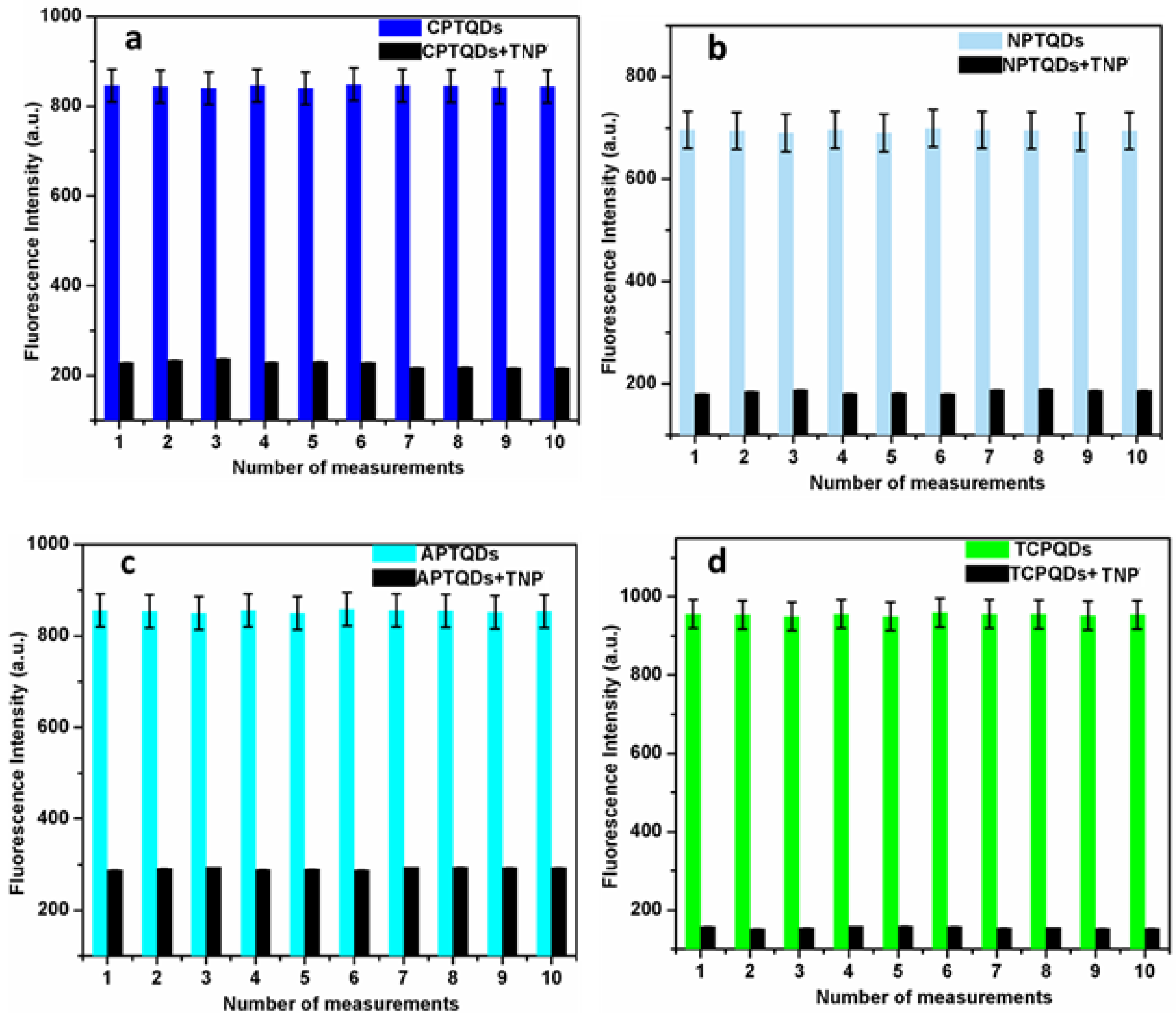


**Figure S21.** Reproducibility of different fabrication series, (a) CPTQDs (in the presence of 0 µM and 7 µM TNP), (b) NPTQDs (in the presence of 0 µM and 20 µM TNP), (c) APTQDs (in the presence of 0 µM and 10 µM TNP), (d) TCPQDs (in the presence of 0 µM and 4 µM TNP). The error bars represent the standard deviation of three measurements.

**References**

[1] B. The Huy, M.-H. Seo, X. Zhang, Y.-I. Lee, Biosens. Bioelectron. **2014**, 57, 310.
[2] S. M. Tawfik, J. Shim, D. Biechele-Speziale, M. Sharipov, Y.-I. Lee, Sens. Actuators, B **2018**, 257, 734.
[3] J. Xue, X. Chen, S. Liu, F. Zheng, L. He, L. Li, J.-J. Zhu, ACS Appl. Mater. Interfaces **2015**, 7, 19126.
[4] X. Peng, Q. Long, H. Li, Y. Zhang, S. Yao, Sens. Actuators, B **2015**, 213, 131.
[5] H. T. Feng, Y. S. Zheng, Chem. Eur. J. **2014**, 20, 195.
[6] N. Dey, S. K. Samanta, S. Bhattacharya, ACS Appl. Mater. Interfaces **2013**, 5, 8394.
[7] A. S. Tanwar, S. Hussain, A. H. Malik, M. A. Afroz, P. K. Iyer, ACS Sens. **2016**, 1, 1070.
[8] G. Yang, W. Hu, H. Xia, G. Zou, Q. Zhang, J. Mater. Chem. A **2014**, 2, 15560.
[9] L. Ding, Y. Bai, Y. Cao, G. Ren, G. J. Blanchard, Y. Fang, Langmuir **2014**, 30, 7645.
[10] V. Bhalla, A. Gupta, M. Kumar, D. S. S. Rao, S. K. Prasad, ACS Appl. Mater. Interfaces **2013**, 5, 672.
[11] X.-H. Zhou, L. Li, H.-H. Li, A. Li, T. Yang, W. Huang, Dalton Trans. **2013**, 42, 12403.
[12] Y. Xu, B. Li, W. Li, J. Zhao, S. Sun, Y. Pang, Chem. Commun. **2013**, 49, 4764.
[13] Y. Wang, Y. Ni, Anal. Chem. **2014**, 86, 7463.
[14] M. Kaur, S. K. Mehta, S. K. Kansal, Spectrochim. Acta, Part A **2017**, 180, 37.
[15] S. Liu, F. Shi, L. Chen, X. Su, Talanta **2013**, 116, 870.
[16] M. Li, H. Liu, X. Ren, Biosens. Bioelectron. **2017**, 89, 899.
[17] H. Ma, C. He, X. Li, O. Ablikim, S. Zhang, M. Zhang, Sens. Actuators, B **2016**, 230, 746.
